# Hierarchical Feedback Modules and Reaction Hubs in Cell Signaling Networks

**DOI:** 10.1371/journal.pone.0125886

**Published:** 2015-05-07

**Authors:** Jianfeng Xu, Yueheng Lan

**Affiliations:** 1 Department of Physics, Tsinghua University, Beijing 100084, China; 2 Collaborative Innovation Center of Quantum Matter, Beijing 100084, China; University of Georgia, UNITED STATES

## Abstract

Despite much effort, identification of modular structures and study of their organizing and functional roles remain a formidable challenge in molecular systems biology, which, however, is essential in reaching a systematic understanding of large-scale cell regulation networks and hence gaining capacity of exerting effective interference to cell activity. Combining graph theoretic methods with available dynamics information, we successfully retrieved multiple feedback modules of three important signaling networks. These feedbacks are structurally arranged in a hierarchical way and dynamically produce layered temporal profiles of output signals. We found that global and local feedbacks act in very different ways and on distinct features of the information flow conveyed by signal transduction but work highly coordinately to implement specific biological functions. The redundancy embodied with multiple signal-relaying channels and feedback controls bestow great robustness and the reaction hubs seated at junctions of different paths announce their paramount importance through exquisite parameter management. The current investigation reveals intriguing general features of the organization of cell signaling networks and their relevance to biological function, which may find interesting applications in analysis, design and control of bio-networks.

## Introduction

With the arrival of the post-genomic era, high-throughput experiments start to provide enormous amount of data to enable a systematic understanding of cell regulation mechanism with unprecedented accuracy. However, we are still lacking of powerful analytic tools to probe how sets of proteins function as an effective machine orchestrating intracellular responses to disparate external stimuli. Take the cancer therapy as an example. Albeit beneficial response is seen upon treatment with the EGFR Tyr kinase inhibitors, the lung tumor often revives by finding ways around, such as inducing a PI3K mutation or activating alternative cancerous signaling pathways [1]. To cope with this type of complication, apparently we need a comprehensive understanding of the global behavior of cell signaling networks shaped through evolution and determined by the involved molecular species and their interactions [2]. Searching for design principles of these highly correlated reaction chains [3, 4] and for critical reaction hubs in complex signaling pathways is an important problem in biological and medical research.

The past decade witnessed much progress on analyzing structures and functions of complex biological networks. Great effort has been devoted to the detection of reaction patterns of diverse gene regulation and metabolic networks [5]. Useful concepts such as the small-world or the scale-free network describe interesting features of these networks from a statistical point of view: the small-worldness narrows the distance between two nodes in a big network while the scale-free network has a power law degree distribution. Various models are analyzed and constructed aiming to offer better understanding of how information is propagated in different networks [6, 7]. Modularity is a more detailed characterization, which, by encapsulating strongly connected chemical species, breaks a system into manageable and functioning pieces. Many interesting modules in complex biological systems are detected with continuous endeavor of researchers, such as the construction of modules in the metabolic network of Escherichia coli [8], the investigation of E. coli transcription regulation network [9], and the identification of modular structure in the Yeast signaling network [10]. Most approaches, however, employ either certain random graph algorithm or some coarse-graining techniques, such as species clustering [5, 11] which puts densely connected vertices to one group and use sparse connections to link different groups. But this type of classification is purely based on network topology and may not be able to retrieve all important features of a biochemical network due to negligence of dynamics information. Recently, probing functions of small recurrent regulation patterns termed network motifs attracts a great deal of attention [12–14]. Functional motifs are believed to constitute major building blocks of biological regulation networks based on which many theoretical predictions are well confirmed by experimental tests [12]. However, complex signaling networks in most cases are not simply built up from these local motifs since even identical motifs may be wired in different manners and function disparately with altered motif dynamics, let alone the fact that different groups of proteins may have complicated and intertwined interactions with each other [15], which may not be captured by a small network with only a few nodes. Moreover, with the rapid development of experimental techniques, the size of networks increases incessantly which brings considerable challenge in recognizing signaling patterns and much difficulty, for instance, in the estimation of kinetic parameters. For all this reason more effective analysis toolkits are called for [15–17].

In this paper, we treat the problem from an information and graph theoretic point of view combined with a consideration of dynamical factors. More specifically, we focus on information flow transmitted from external cues to target proteins through forward signaling pipelines adjusted by miscellaneous feedback modules [18]. Here is a brief description of our algorithm. By constructing a directed graph based on all relevant biochemical reactions, we are able to search a set of shortest cycles covering almost all the edges. By recursively searching for intersections of these cycles with paths linking the input to the output we collect all the paths belonging to the forward module and hence extract feedbacks from the rest nodes. Besides the network topology, dynamics information is taken into account such as the initial molecular concentration, the necessary condition for the information to pass the binary reactions. In this way a signaling network is decomposed into functionally different parts, through which the detailed regulation mechanism is much more easily seen. As shown by our results, together with forward signaling paths, the feedback loops make up modules of different scales and types: local and global, positive and negative. It should be emphasized that precise values of chemical kinetic parameters are not required in our decomposition, which suggests the direct applicability of the current scheme to experimentally identified signaling networks which have not received much quantitative characterization.

We applied our method to quite a few signaling systems among which three are presented here: the G protein coupled receptor (GPCR) mediated calcium spark network [19–22], the EGFR induced MAPK signaling network [23–25], and the JAK/STAT signaling network [26, 27]. Forward signaling modules and feedback control modules are automatically retrieved and defined in each of these networks, giving new insights into the organization and functional roles of different chemical species in a network. Forward modules contains multiple channels and usually constitute a self-sustainable part of the network while feedbacks modulate signaling in a hierarchical way and on different features. The function of individual module could be checked with the module perturbation technique: deterministic computation of target protein concentration with deletion of individual feedback modules, one at a time from the full network. The obtained results show that local and global feedback controls are dynamically layered both in function and in time. Furthermore, plausible explanation is given on why local feedbacks are well conserved while global ones are more miscellaneous based on their dynamics heterogeneity, which partially rationalizes the idea of network motif. Sensitivity analysis reveals bifurcation in signaling dynamics, which on one hand detects key kinetic parameters associated to reaction hubs and on the other hand suggests possible sudden change of dynamical behavior upon parameter variation. Our decomposition procedure is able to provide a clear signaling map of information flow and reaction hubs, depicting key connections between feedback modules and disclosing structural basis of signaling systems’ functional redundancy in light of capabilities of different biological components [28].

## Results

### Skeletons of signaling networks are conveniently identified by retrieving forward and backward information flows

**Fig 1:**
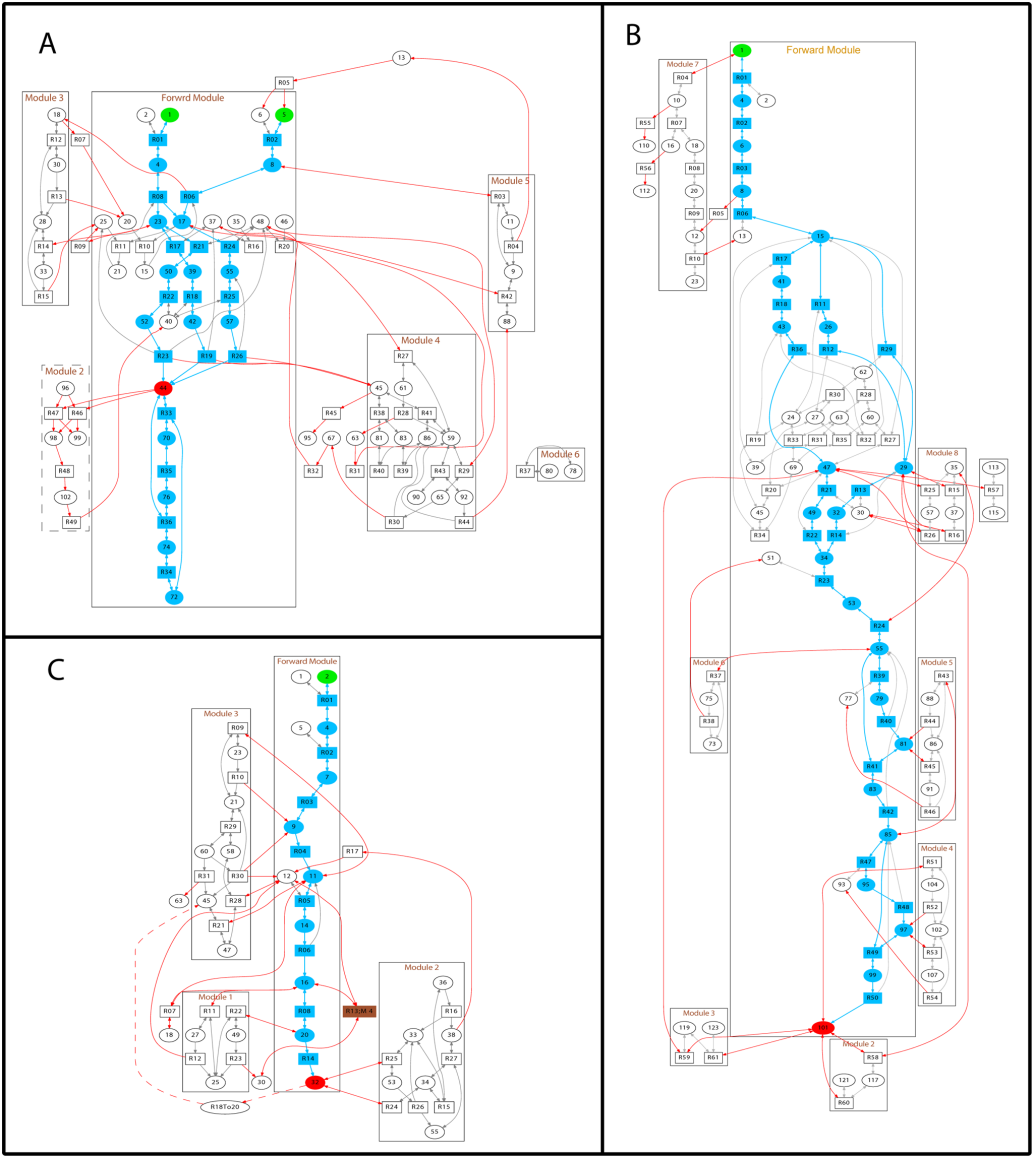
The decomposition of the GPCR, the MAPK and the JAK/STAT signaling system. (A) The decomposition result of the GPCR mediated IP3-Ca^2+^ signal transduction pathway. One signaling forward module and four feedback modules are plotted. Green nodes are inputs of the networks, while red ones represent outputs; ovals symbolize reactants and products and squares functional nodes representing specific reactions. The edges connecting ovals to squares associate the participants to each reaction. In addition, a bi-directional line indicates that the reaction is reversible. (B) The decomposition result of the MAPK signaling network. The full network is decomposed into one big forward module, and seven feedback modules. (C) The decomposition result of the JAK/STAT signaling network. One main route constitutes the forward module. Module 1 and 2 are mid-ranged feedback controllers. A local feedback loop and the global gene regulation feedback module are combined in Module 3.

It is commonly believed that cellular multitasking responses to various extracellular stimuli are attributed to complex signaling regulation. The study of information flow in cell signaling helps unravel the relationship between structure and function of these networks. Our strategy is to decompose signaling pathways into information forwarding pipelines and feedback loops, which turns out to be an efficient way to determine signaling modules. We apply our method to three signaling networks: the GPCR signaling networks, the MAPK signaling system and the JAK/STAT signaling pathway. These systems are chosen since they are activated by three different kinds of motifs capturing the majority of signaling stimuli and, in most cases, they coexist in the same cell cross-talking to each other. Moreover, the JAK-STAT signaling networks contains gene regulation. Common rules shared by these systems are very likely to apply to other signaling networks. We find that they all consist of a signal-relaying module which transmits signals quickly from the input to output and several signal-modulating modules which control detailed temporal profile of signals (Fig 1), although the network topology and underlying chemical mechanisms are vastly different.

**Fig 2:**
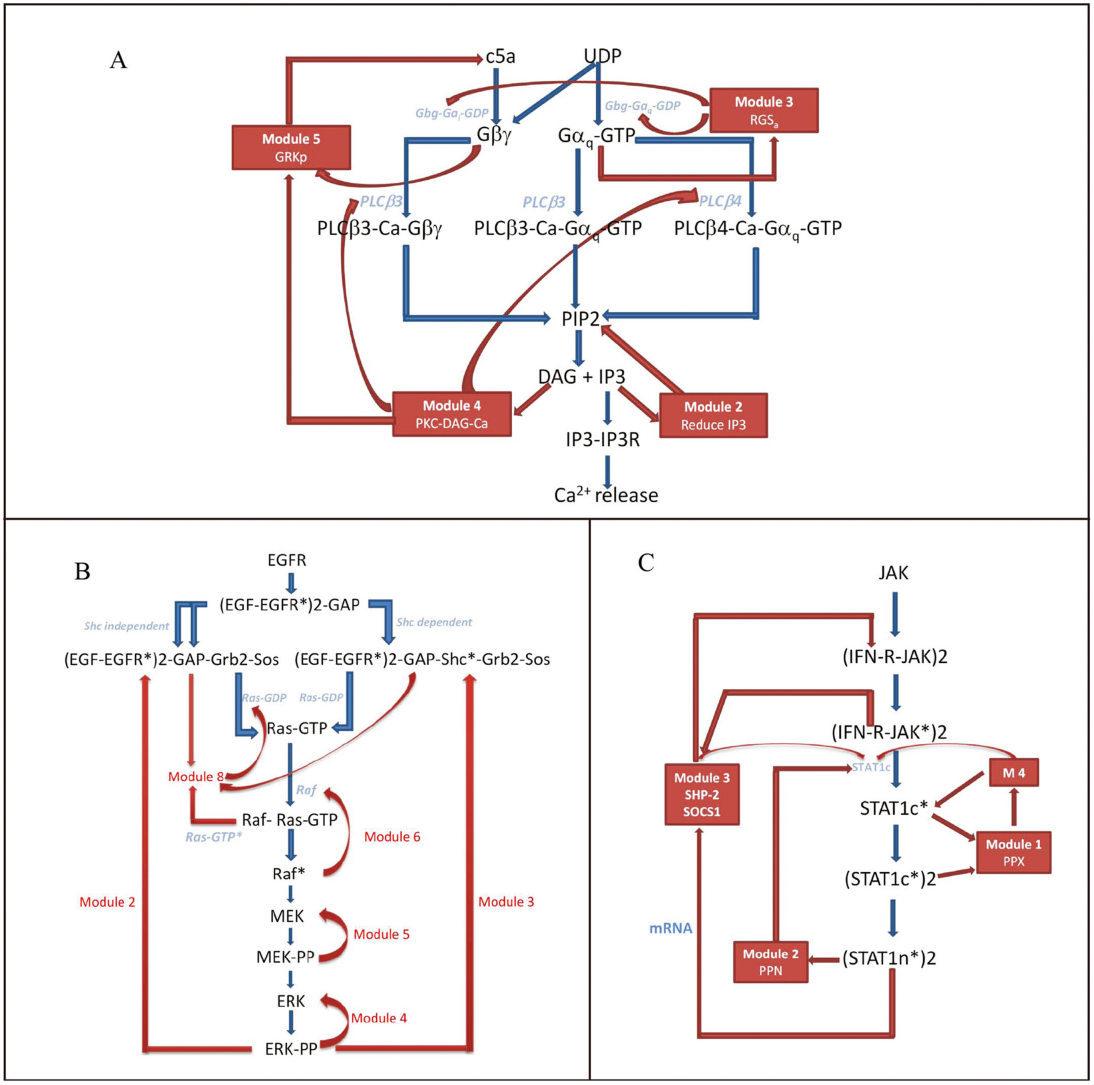
The Simplified schematics of the three signaling networks. (A) The sketch is related to the GPCR signaling pathway. The pathway in blue are the skeleton of the forward module depicting how signals propagate forward. Feedback modules in red indicate multiple regulations. The RGS in Module 3 drive two proteins: G*α*i-GTP and G*α*q-GTP into the off state: G*α*i-GDP and G*α*q-GDP. Reactions in Module 2 constitute a reaction chain, that is: IP3—> IP4—> IP5—> PIP2, and hence reduce the concentration of IP3. Module 2 and 3 are local feedbacks while Module 4 and 5 are working together serving as a global feedback module (B) The skeleton of the EGFR induced MAPK signaling network. Module 4, 5, 6 are three joint dephosphorylation feedback cycles. Module 4 is engaged in the dephosphorylation of ERK-PP, while Module 5 and 6 are able to convert MEK-PP and Raf* to MEK and Raf. Module 8 is also a local feedback serving as a buffer for the highly regulated compounds: (EGF-EGFR*)_2_-GAP-Grb2-Sos, (EGF-EGFR*)_2_-GAP-Shc*-Grb2-Sos. It also takes in the upstream protein Ras-GTP* and feeds the Ras-GDP back. Two global feedback modules reside near the end of the network. (C) A simplified plot of the JAK/STAT signaling network. The binding of the IFN-*γ* to its receptor causes activation of the transcription factor (STAT1n*)_2_. The expression product SOCS1 suppresses signaling. SHP-2, serving as a tyrosine phosphatase in Module 3, can directly sequester the activation of (IFN-R-JAK*)_2_. PPN (nuclear phosphatase TC45) involved in Module 2, and PPX (cytoplasmic phosphatase) embedded in Module 1 act in the same way to capture (STAT1n*)_2_ or (STAT1c*)_2_ and feed the inactive STAT1 back to the forward module.

The paths marked blue in the forward module of each full signaling network in Fig 1, constitute a subgraph which is termed the most efficient production unit (MEPU). The MEPU is a set of paths passing signal from the input to the output with minimal number of nodes and complete reaction components and shores up the whole forward module. The way of getting MEPU is described in Materials and Methods. Based on the MEPU, essential and ordered signaling events in the forward module are identified and detailed controls by feedback modules are analyzed. For convenience, a schematic diagram for each signaling system (Fig 2) is plotted with key proteins drawn from the decomposition results (Fig 1). The key proteins or so-called reaction hubs at which the reaction paths split or converge in the MEPU are linked by green arrows depicting the forward signaling while those interacting with feedbacks are marked with feedback loops, constituting a highly simplified flow charts of the network.

Compared to the undisposed GPCR network (S1 Fig) based on the model proposed by Flaherty *et al*[19], the decomposition result in Fig 1A gives a clear picture of how information flows forward and how feedback controls the output (reactants or products corresponding to the round nodes are listed in S1 Table and chemical reactions for square reaction nodes can be found in S3 Table). As depicted in the schematic plot (Fig 2A), G*α*-GTP and G*βγ* are able to bind to the isoforms of PLC including PLC*β*3 and PLC*β*4, catalyzing synthesis of IP3 and DAG out of PIP2. Apart from the forward module, the feedback modules in red exert multiple regulations to different parts of the network. Here we take the EGFR mediated MAPK signaling model (S2 Fig) proposed by Hornberg *et al*[23] (reactants are listed in S4 Table and chemical reactions are in S6 Table). As can be seen in Fig 2B, dimeric EGFR recruits GAP, Sos, Grb2 in the Shc-dependent and independent routes. Then, Sos brings the compound close to the membrane-anchored Ras. The interaction between Sos and Ras results in the activation of Ras-GTP which initiates the MAPK cascade through three phosphorylation events and finally produces the target protein, ERK-PP. Most feedback modules reside near the end of the network. Among the JAK and STAT families, Janus kinase connected with IFN-*γ* via STAT1 is ubiquitous throughout different cell types [29]. We analyze the model proposed by Yamada *et al*[26] (reactants are listed in S7 Table and chemical reactions are in S9 Table). It is easy to check the major signaling events in Fig 2C. Binding of the IFN-*γ* to its receptor causes dimerization of the receptor and then activates the JAK association. The compound is in turn phosphorylated by the activated JAKs into the form (IFN-R-JAK*)_2_, providing a docking sites for STAT1 in cytoplasm (STAT1c). STAT1c is then activated by JAK and dimerizes (STAT1c*). The dimerized (STAT1c*)_2_ then leaves cell membrane and translocates to the nucleus to initialize gene transcription. In contrast to the GPCR and the MAPK networks, the JAK/STAT network as shown in Fig 2C contains only one channel in the forward module and signaling is regulated by gene transcripts. Yet, feedback modules are notable from the input all the way down to the output.

### Hierarchical structure of feedbacks is clearly revealed in the decomposed network

Cell signaling networks achieve effective life activity control via various feedback loops that connect output signals back to the inputs [30]. They are indispensable in maintaining cellular homeostasis [31] and making liable cellular decisions [13]. Together with forward signaling paths, the feedbacks make up modules of different scales and types: local and global, positive and negative. The global feedbacks are feedback loops linking the output to the input and the local feedbacks are those wrapped by global ones. However, for signaling systems without global feedbacks, then feedbacks wrapping the local ones in the outermost layer are taken as global feedbacks. Moreover, negative feedbacks are those counteracts the forward signaling and positive ones otherwise. It turns out that such modules are structured in a hierarchical way in all three signaling networks, which provides structural evidence of layered design of these networks.

Based on the decomposition of the above signaling networks, we find that local feedbacks are distributed heterogeneously along the pathway and coupled with fast signaling procedures in the forward module. As depicted in Fig 2A, two local feedback modules are detected in the GPCR signaling network. Module 3 contains the regulators of G protein signaling (RGS) that increase the hydrolysis rate of GTP [32]. The RGS proteins remarkably reduce the lifespan of the GTP-bound molecular complex GTP-G*α*i (N18) and GTP-G*α*q (N23). The deactivated GDP-G*α*i (N20) and GDP-G*α*q (N25) are sequentially turned into G*βγ*-G*α*i-GDP (N15) and G*βγ*-G*α*q-GDP (N21) making two feedback cycles. Another local feedback: Module 2, is related to the Ca^2+^ inducer: IP3. In this feedback loop, IP3 changes to IP4 [33] and then to IP5. IP5 finally converts to PIP2. This feedback loop not only directly reduces the concentration of IP3 but also feeds signals back to the upstream since PIP2 is the input of the Reaction 22, 18 and 25. Analogously, this feedback is coupled with fast forward reactions since the binding of IP3 to the IP3R greatly enhances the outflow of calcium ions from the endoplasmic reticulum. Unlike the RGS protein, this feedback loop splits the signal and temporally transforms the regulated IP3 to other chemical species. As for the MAPK signaling (Fig 2B), combining the phosphorylation cascades induced by Raf, MEK, and ERK, Module 4, 5 and 6 generate three joint dephosphorylation feedback cycles [34], one followed by another. Since the MAPK signaling cascade is known for quick amplification of upstream signals, these local feedbacks keep the target protein from being persistently activated. Another local feedback module in the upstream: Module 8, acts in a fast process to absorb the Ras-GTP* from the forward module and feeds the Ras-GDP back. There is also one local feedback loop in the JAK/STAT signaling network (Fig 2C). SHP-2, serving as a tyrosine phosphatase in Module 3, can directly suppress the activation of (IFN-R-JAK*)_2_ which is also fast compared to other reactions in the JAK/STAT signaling networks.

On the other hand, global feedback modules constitute a relatively large portion of all feedback modules and tend to wrap local ones resulting in a hierarchical structure. Compared to the local feedbacks, the organization of these global feedback modules varies from network to network since they employ more functional proteins. They may cooperate to serve one regulation. For example, the two modules: Module 4 and Module 5 in the GPCR network are linked together to form a global one (Fig 2A). The feedback signal from Module 4 is passed on to Module 5 resulting in a long-range feedback loop. Global feedbacks occasionally cooperate with local modules to implement effective control. With regard to Module 3 in the JAK/STAT signaling networks (Fig 2C), the association of gene product SOCS1 with (IFN-R-JAK*)_2_ and the subsequent binding with STAT1c makes up a global feedback module. The incorporation of protein SHP-2 into SOCS1-(IFN-R-JAK*)_2_-STAT1c gives rise to a crosstalk between the local and global feedback loops. Global feedback modules also work coordinately. ERK-PP, the target of the MAPK system (Fig 2B), directly participates in the reactions of feedback Module 2 and 3. These feedback modules are working in similar manners to buffer free ERK-PP. Another example is depicted in the schematic plot of the JAK/STAT signaling in Fig 2C. PPN (nuclear phosphatase TC45) involved in Module 2, and PPX (cytoplasmic phosphatase) embedded in Module 1 act in similar ways to absorb (STAT1*)_2_ and feed the inactive STAT1 back to the forward module. However, unlike the two global feedback modules in the MAPK pathways, these feedback modules act at different sites inside the cell: PPN in the nucleus while PPX in the cytoplasm. Feedback modules serving as a buffer pool are prevalent in all three signal networks and, in most cases, are negative regulators since they usually act to reduce signals and the transformed output molecules probably move off for other purposes.

### Local and global feedback control are dynamically layered both in function and in time upon module perturbation

**Fig 3:**
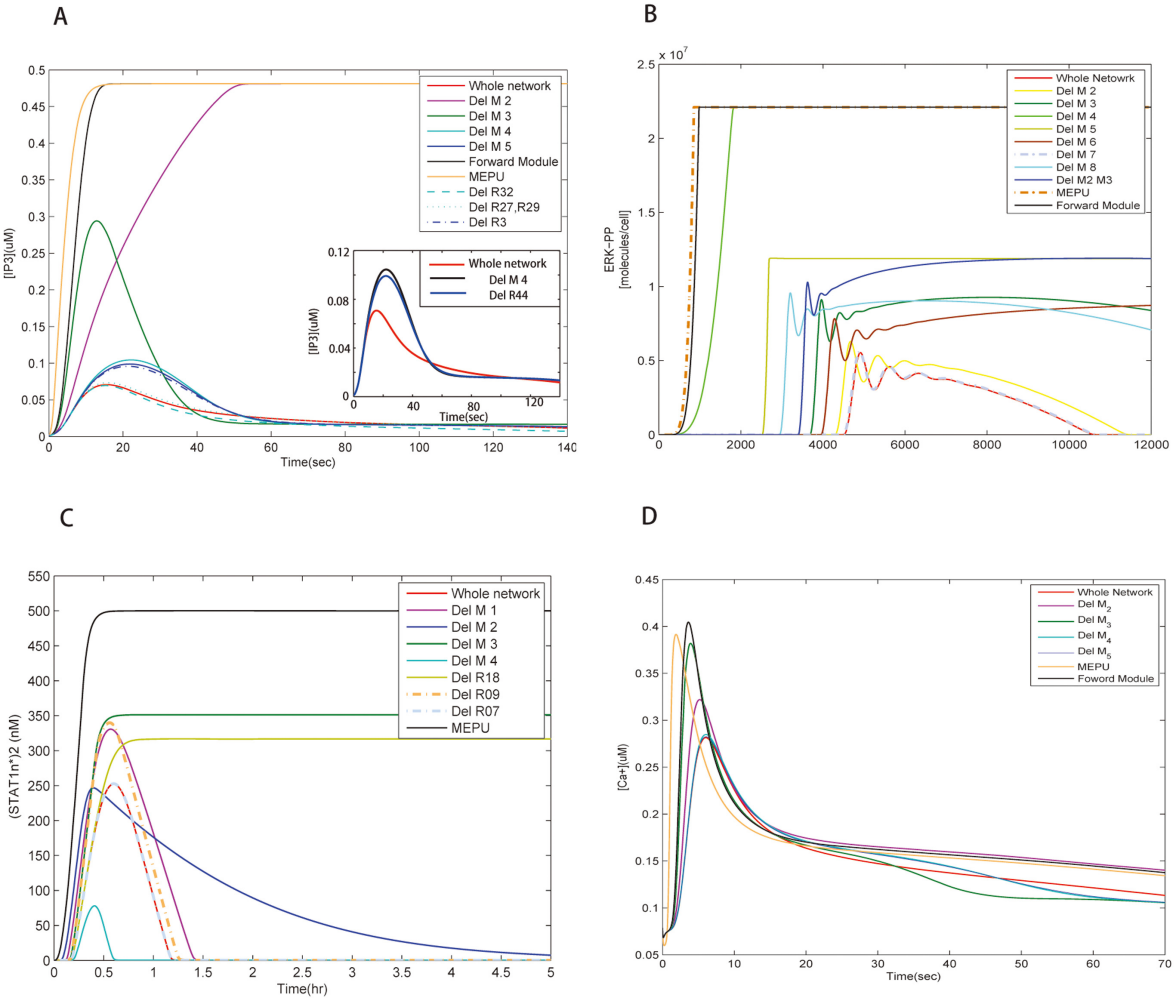
The dynamical behavior of the target protein in the three signaling systems. (A) The IP3 concentration of the GPCR pathway over time by deleting individual feedback modules from the full network. All feedback modules in red act at different time scales and in different ways. (B) The ERK-PP concentration over time by deleting individual feedback modules from the whole MAPK network. Different feedback modules carry out different but synergetic functional duties during cell signaling. (C) The change of the (STAT1n*)_2_ concentration over time by deleting individual feedbacks from the whole JAK/STAT network. The upstream local feedback (R09) delays the response significantly, and the feedback module (Module 3) directly linked to the target attenuates the output signal. (D) The change of Ca^2+^ concentration over time in the GPCR signaling system. Note that the faster the increase of [IP3], the shorter the response time and the higher the amplitude of the Ca^2+^ spark. A quick time response of IP3 determines the profile of the Ca^2+^ spark; and the amplitude of IP3 concentration does not seem to be of primary importance.

Feedback loops are obbligato elements to maintain cellular functions in handling internal and external stimuli and perturbations [35]. Different modules of decomposed signaling networks give us the convenience in systematically probing dynamics of each functional unit by module perturbation. From the simulation results (Fig 3), we observe that the behavior of target proteins is more vulnerable to the deletion of local feedbacks than of global ones. That is, local feedback modules exert strong influences on signaling while global feedbacks work in subtle ways. Different local feedbacks seem to have different influence on the amplitude and delay of the output signal, which generates layered structures in the temporal profiles of the output.

The dynamics of IP3 in the GPCR signaling network is shown in Fig 3A in which we find that local feedbacks are dominant and global ones finely adjust the concentration of the target protein. From the purple curve, we notice that removing the local feedback Module 2 changes the output signal dramatically: the system maintains high concentration of IP3 after a relatively long transition period. Such dramatic change indicates that Module 2 plays a crucial role in reducing the concentration of IP3. As previously mentioned, in this module, IP3 changes to IP4 and then to IP5, which is largely accumulated in Module 2 due to its small conversion rate to PIP2 and hence brings down [IP3] considerably. Unlike Module 2, another local one–Module 3 regulates the signaling at the upstream of the network. When we remove this module, the concentration of IP3 increases rapidly before attenuation, even faster than when removing Module 2 since Module 3 is a upstream regulator. Evidence shows that mutations inhibiting the GTPase activity are correlated with a significant portion of human tumors [36], probably due to full or partial loss of signaling control at the beginning of many pathways. With regard to global feedbacks, when we delete either Module 4 or Module 5 from the full network, a modest rise of the output signal results. There are also links between Module 4 and the forward module. Whereas, the removal of these links from the network, such as R27, R29, R32 (Fig 1A), has little effect on the output signal (Fig 3A) exhibiting the network’s resilience to non-essential change. Being not so important in rapidly reducing signals, global feedbacks are significant in diversifying signaling dynamics. As depicted by the green dotted curve (Fig 3A), the removal of R32 connecting Module 4 lowers the concentration of IP3 indicating that feeding PLC*β*3-Ca back to the forward module is a positive regulation. The simulation also manifests that possessing more functional proteins bestows global feedbacks varied measures to affect the output. For example, the deletion of reaction 44 (R44) connecting Module 4 and 5 has a similar effect on the output. These observations indicate synergy between activity of the two modules.

By simulating the concentration of ERK-PP in the MAPK signaling network (Fig 3B), we see that local feedbacks not only reduce the signal but also generate oscillation while global ones modulate fine features of the oscillatory behavior. Local modules share a common block of biochemical process: the MAPK cascade, whereas they play different roles in signal regulation. At the bottom, Module 4 and 5 modulate the dynamics in an essential way since deleting either of them results in the disappearance of oscillation and the final damping which, in turn, reflects the high sensitivity of the MAPK cascade [37, 38]. As we know that one way to trigger oscillation is to attach a time-delayed negative feedback, the interaction of the MAPK cascade with these feedbacks is the cause of oscillation. Moreover, two steady states are observed in this system when deleting module 4 and 5 which might relate to the fact that the MAPK system is able to maintain disparate stable concentrations concerning its multiple functions coping with different extra- or intra-cellular stimuli [39]. Module 6, situated slightly further from the output node, also plays a vital role in bringing the signal down to the static level. Module 8 is placed at the upstream to attenuate and control delay of signaling since deletion of this module advances the signaling response. However, the local feedbacks are not uniform in reducing the signal and it seems that the nearer to the target, the stronger a module’s influence. The remaining modules are global feedbacks. Two long feedback edges emanate from Module 2 and 3, serving as buffers of ERK-PP and modifying the output in a more gentle way. These global feedbacks announce their importance in *in vivo* experiments because they lead to different behavior of ERK [40]. In this case, they seem to prolong signal oscillation since the deletion of Module 2 or 3 shortens the oscillation (Fig 3B). The MAPK’s oscillatory dynamics plays important roles which is confirmed experimentally. For example, the sustained oscillation of MAPK leads to the corresponding oscillations in the yeast mating-gene expression [41] and the rapid nuclear-cytoplasmic ERK oscillations are observed in human mammary epithelial cells [42]. Yet, the two global feedbacks behave similarly but not identically. Simulation results show that Module 3 is relatively effective in lowering [ERK-PP]. The Shc dependent pathway plays a predominant role in the MAPK cascade [38] since the protein Shc improves the affinity between (EGF-EGFR*)_2_-GAP and Grb2. So deleting Module 3 where Shc lies has much influence on ERK-PP signaling. Interestingly, based on the simulation, we find that the activation time determines the amplitude of the signal: the earlier the activation, the larger the amplitude. The amplitude and delay locking mechanism is also found in other signaling pathways [36] and is designed for reliable cell decision making.

Unlike previous two systems, the JAK/STAT signaling network involves gene expression regulation. Among the identified four feedback modules, Module 3 has significant influence on the output since removing this module disables the system in bringing down the concentration of (STAT1n*)_2_. There are two regulators related to Module 3: a global feedback involving the SOCS1 protein and the SH-P related local feedback control. As previously mentioned, the global and local regulators are intertwined to adjust the dynamics patterns. From the simulation result shown in Fig 3C, we see that the dynamics after removing Module 3 can be divided into two parts. The first corresponds to the dynamics in the absence of gene regulation as depicted by the yellow curve since deleting R18 blocks transcription. Another part is related to the absence of R09 which directly impedes the inactivation of (IFN-R-JAK*)_2_. The clear identification of different dynamics originated from local and global feedbacks may give us clues for efficiently designing controllable biological systems. Two global feedbacks: Module 1 and 2 are both the buffers of (STAT1*)_2_. The binding of PPN to (STAT1n*)_2_ in Module 2 decreases (STAT1n*)_2_, which as a result directly attenuates the output signal. With the aid of PPN, the inactivated STAT1c is fed back to the forward module. Although the amplitude is not affected the rapid decline of [(STAT1n*)_2_] is alleviated by the removal of Module 2, which implies that Module 2 is engaged in the quick attenuation of the output. The concentration of (STAT1n*)_2_ is reduced to about one fourth after we knock out Module 4, which is explained by the fact that the absorbed STAT1c* enters Module 1 and gets back to the forward flow as STAT1c through Module 4. Blocking this channel cuts down output signals. Since Module 1 absorbs (STAT1c*)_2_ in the cytoplasm but not directly affects the (STAT1n*)_2_ in the nucleus, the dynamics are more influenced by Module 2 indicating that if different controllers act on the same biochemical pathway, the ones closer to the target have more influence.

### Multiple information channels are identified automatically which ensure functional redundancy and dynamical stability of signaling

Functional redundancy or structural degeneracy is closely related to robustness of biological systems [28] since the ensured stability increases the survival rate of cell in face with catastrophic changes in the environment or induced by gene mutation. From the decomposed network, identifying this redundancy becomes an easy job. Based on the observation and comparison of different signaling pathways, we found that cells tend to employ different redundancy mechanisms throughout the whole network to ensure the robustness of dynamics.

Feedback modules employ three ways to achieve functional redundancy in the three signaling networks. The most obvious one is the feedback module attached to the same protein with the same regulating mechanism, such as the two global feedback modules in the MAPK system (Fig 2B) and those directly absorbing (STAT*)_2_ in the JAK/STAT system (Fig 2C). Secondly, feedback modules may sequentially and cooperatively achieve one function to minimize the noise influence. Removing any module from the full system generates similar variation of output signals. For instance, the global feedback loop in the GPCR pathway is composed of two coupled feedback modules (Module 4 and 5 in Fig 2A). Deleting either one causes same dynamical changes in the output signal. Lastly, two modules with different targets and control schemes may have similar effect on signaling. For example removing Module 1 from the JAK/STAT signaling network has similar effect to the removal of the local feedback controlling SHP-2 (the curve related to the deletion of R09) as shown in Fig 3C.

**Fig 4:**
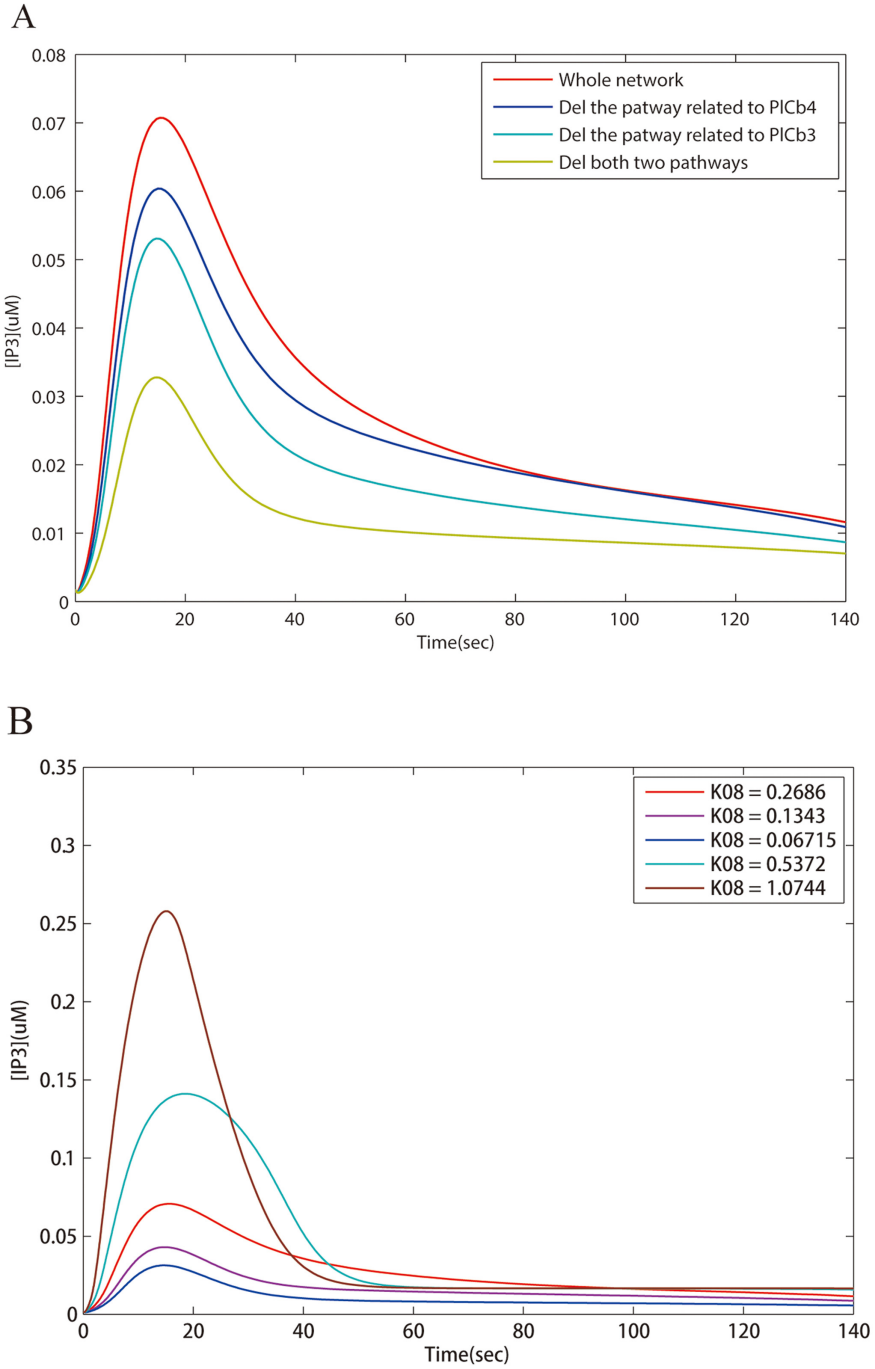
The functional redundancy test and the parameter sensitivity analysis of crucial proteins in the GPCR signaling network. (A) Functional redundancy in the forward module of the GPCR signaling network. Two pathways being PLC*β*3 or PLC*β*4 dependent, fork at G*α*q-GTP and converge at IP3. Their function shows redundancy since the concentration of IP3 still reaches as much as 80 percent of the original amplitude even if removing one of them from the signaling network. But when they are both removed, signal only reaches half of the original amplitude indicating the complementarity between the two paths in conducting signals. (B) Sensitivity to parameters of the reaction hub in the forward module of the GPCR signaling network. The reaction rate K08 is the synthetic rate of G*α*q-GTP and G*βγ*. Both products are essential for signal forwarding.

**Fig 5:**
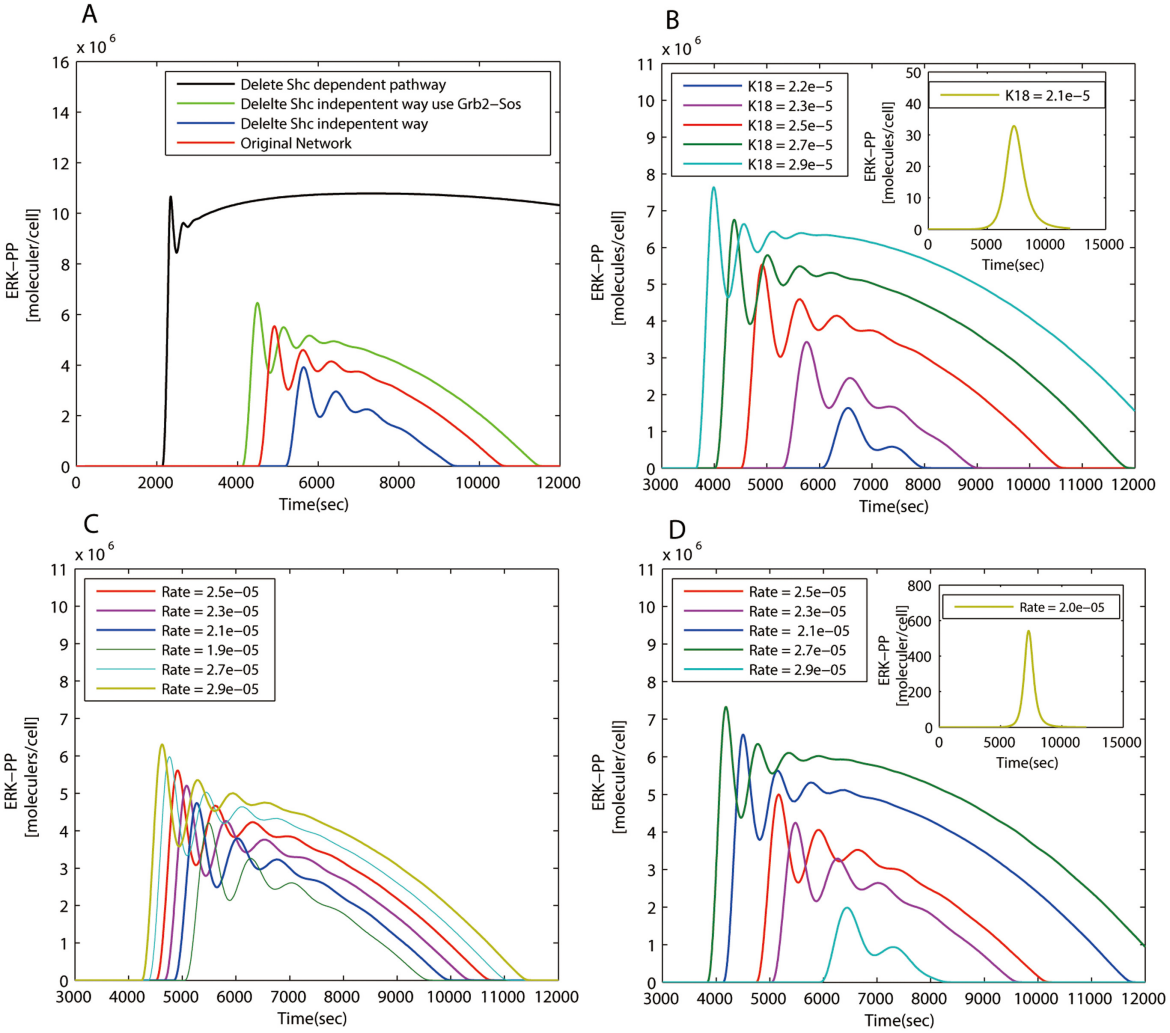
The redundancy test and the parameter sensitivity analysis of the MAPK signaling networks. (A) Two pathways from (EGF-EGFR*)_2_-GAP to (EGF-EGFR*)_2_-GAP-Grb2-Sos are both Shc independent. The blue and green curves indicate that removing either path has similar impact on the concentration of ERK-PP. (B) The sensitivity analysis for the reaction generating (EGF-EGFR*)_2_-GAP. A small change in the reaction rate has a big effect on the output signal. A bifurcation is observed when k18 is less than 2.1e-5. (C) Sensitivity analysis of the binding rate of Ras-GDP to (EGF-EGFR*)_2_-GAP-Grb2-Sos. (D) Sensitivity analysis of the binding rate of Ras-GDP to (EGF-EGFR*)_2_-GAP-Shc*-Grb2-Sos. Another bifurcation is observed at the rate 2.0e-0.5.

On the other hand, multiple signaling paths and crosstalks in the forward module bestow extra robustness to a signaling network. With regard to the inherent complexity of signaling networks, such as multiple input-output pairs and functional diversity of hub proteins, it is not totally out of expectation that different signaling channels exist in the forward module. In the forward module, the MEPU is the most sensitive part in response to extracellular stimuli which can be observed in Fig 3. So the MEPU is helpful in searching for multiple paths with similar signaling functions. As depicted in Fig 2A, two pathways in the GPCR network fork at G*α*q-GTP and converge at IP3. They have similar functions since the concentration of IP3 reaches as much as 80 percent of the original amplitude by removing either from the signaling network as shown in Fig 4A. But when they are removed together, only half of the amplitude is reached, indicating the complementarity between the pathways in conducting signals. Analogously, the MAPK signaling network also possesses multiple signaling channels in the forward module. From Fig 2B, two pathways from (EGF-EGFR*)_2_-GAP to (EGF-EGFR*)_2_-GAP-Grb2-Sos are both Shc independent and serve similar functions as shown in Fig 5A. Additionally, two main pathways activate Ras-GTP, one of which is Shc dependent and functionally redundant. Notably, in the MAPK system, even in the absence of a number of reactions in the forward module, the behavior of the MEPU and the full forward module does not change significantly suggesting functional robustness in signal forwarding. The paths excluded form the MEPU is redundant in the forward module, which are obviously and conveniently disclosed in our procedure.

### The reaction hubs sitting at path junction are tightly regulated and bifurcations are observed upon parameter variation

The clean decomposition of a network enables looking in great detail into biochemical information flows and answering questions like: how reaction chains transduce external stimuli to the target, and how feedbacks efficiently regulate concentration of specific products. Answers to these questions help us identify reaction hubs in signal transduction. The reaction hub is an essential node to transmit information in a signaling network, which also facilitates crosstalks between modules and collect signals from different channels to fine-tune the response to stimuli [43]. Our analysis shows these critical nodes usually sit at junctions of different paths and are highly regulated, which make up the core nodes of a signaling network and support the overall information transmission routes.

Multi-input and multi-output networks give rise to complicated crosstalks between modules. By finding out the MEPU in the forward module, one can clearly identify reaction hubs since it is easy to check at which node two or more reaction paths converge and through which reaction or reactant the chemical reaction chain splits. In most cases reactants at junctions where scaffold proteins likely live, are hubs for controlling information flow [44]. For example, Ras-GTP collecting multiple stimuli from upstream pathways to activate Raf is crucial for downward signaling since the activated Raf is essential for the MAPK cascade. While in the GPCR signaling, the convergence of information flow indicates that the cell membrane protein PIP2 serves as an information collector. Expectedly, proteins in the forward module of the JAK/STAT signaling network are all crucial since deleting any node from the MEPU blocks signaling. On the other hand, proteins at which signaling paths split are also important. Fig 5B shows the sensitivity analysis of one parameter that controls the synthesis of (EGF-EGFR*)_2_-GAP, which acts as a reaction hub since three pathways leading to the target protein originate from this compound. It is easy to see that [ERK-PP] is very sensitive to the value of this kinetic parameter. A slight increase or decrease of the parameter affects the concentration of ERK-PP to a large extent. Observably, when the parameter is a quarter of the original value, the production of ERK-PP is dramatically decreased indicating possible existence of a threshold of activation. Analogously, in the GPCR signaling network, the reaction that transforms G*βγ*-G*α*q-GDP to G*α*q-GTP and G*βγ* is a reaction hub. And sensitivity analysis (Fig 4B) shows that a change in the reaction rate has a prominent influence on the output.

The signaling skeletons of the three schematic plots (Fig 2) were obtained by marking and linking critical nodes in the decomposition results. Besides the reaction hubs in the forward module, those connecting the forward and feedback modules or being at a joint between different feedback modules are, intuitively, important in regulating signals. For instance, STAT1c in the JAK/STAT signaling is not only an indispensable element for signal relay but also an essential hub for signal modulation since it is regulated by multiple feedback modules. Removing one of the controllers, reaction 13 (Module 4) significantly weakens the cellular signal since the back-flow of STAT1c is blocked. On the other hand, impeding the transcription of (STAT1n*)_2_ leads to failure in damping the output signal (Fig 3C) which highlights the role of SOCS1, a joint between the transcription module and feedback Module 3. For the GPCR signaling network, signaling behavior (Fig 3A) when the reaction (R44) between feedback Module 4 and 5 is deleted is similar to that of deleting the whole related feedback modules, indicating that this reaction is the primary information gate between these modules. In the MAPK signaling network, two reaction hubs: (EGF-EGFR*)_2_-GAP-Grb2-Sos and (EGF-EGFR*)_2_-GAP-Shc*-Grb2-Sos are also regulated by more than one feedback modules. As depicted in Fig 5C and 5D, the concentration of ERK-PP is sensitive to alteration of reaction rates related to these two compounds. Comparatively, the kinetic parameter associated to (EGF-EGFR*)_2_-GAP-Shc*-Grb2-Sos are more effective in controlling the output indicating that the Shc dependent and independent pathways are not functionally equivalent. It is also proved in simulation as shown in Fig 5A that the absence of the Shc dependent pathway brings about a dramatic change in signal strength, delay and decay rate since the deletion of this bypass disables the influential global feedback Module 3. Henceforth, we conclude that the bifurcation presented in Fig 5B and 5D is related to the Shc dependent pathway.

## Discussion

We apply our decomposition procedure to three typical signaling networks: the GPCR mediated calcium spark network, the EGFR induced MAPK signaling network, and the JAK/STAT signaling network to acquire a quantitative and systematic understanding of the underlying organizing principles. Several common features are recognized that could be ubiquitous in cell signaling since the three signaling networks employ different types of receptors inducing diverse signaling pathways and play distinct yet important roles in living cells. The results show that all three signaling networks are decomposed into one signal forward subgraph and several local or global feedback modules, which brings great convenience and provide new means in analyzing correlation between network structure and their individual function.

Feedback modules in cell signaling networks are organized in an intricate yet controlled way. Local feedbacks are usually coupled with fast signaling procedures in the forward module in a straightforward manner. Global feedbacks are structured and behave in much more diverse ways, not only in organizations, but also in functions. From our computation it seems that global feedbacks are closely related to crosstalks since they employ various multi-functional proteins. Although the JAK/STAT pathway we discussed here is not overly complicated, the components in this system closely correlate to different signaling pathways, such as the MAPK signaling, the PI3K signaling and many others [45]. The GPCR pathway also mediates the activation of the EGFR signaling [22]. The three signaling networks employed in this paper work coherently in one cell and could in principle be combined and subject to analysis in one big frame where global feedbacks may announce their importance in bridging functional connections. One interesting observation is that a major portion of feedback modules sit near the output of a signaling system. That is, downstream proteins or targets of networks are well regulated. This designing scheme facilitates careful and in-time control of output signals. Apart from the downstream regulation, in all three signaling networks, there is a local feedback module in the upstream region being a safe guard negatively affecting downstream signals by switching off onset proteins. As we know, protein’s switching off is as important as switching on in cell signaling since activated molecules should return to its original resting state so as to prepare for the next signaling event [46]. Moreover, we see no feedbacks cross each other in the decomposed graph and feedbacks always show up layer by layer. One explanation is that cell signaling networks shaped by environmental changes may acquire finer and more versatile modulation of signals during evolution. Wrapping additional layers of control loops around existing ones is reasonable since it may be harmful and inefficient to rewire all the connections in a well-functioning module. As a result, more and more complicated signaling structures are generated, yet, in a hierarchical way.

Local and global feedbacks work together to shape the behavior of the whole signaling network. The simulation results show that dynamical behavior of target proteins is modulated by the detailed control associated to each module. For example, in the GPCR signaling system, Module 2 brings down the signal, but acts during a relatively long time interval. Module 3 suppresses the signal most efficiently; Module 4 and 5 act at a time scale in between. Different time scales of regulations keep the concentration of IP3 at an acceptable level and with a characteristic temporal profile. However, signaling behavior with strong local feedback modules being removed changes much more than when removing global feedbacks, which indicates that local feedback modules are associated with strong regulation and global feedbacks subtly control output signals. It is a good strategy for the strong local negative feedbacks to maintain homeostasis since abnormally high concentration of signaling targets may be toxic to cell and could also be a waste of energy and resources. For extra fine control, multiple long-ranged feedbacks are needed to tenderly trigger or gently inhibit signaling. The cooperation between feedback modules is essential for the robustness of biological systems and acclimates cells to assorted environments and random non-crucial mutations. It also contributes to the functional versatility of signaling networks. For example, in the MAPK system, the local feedbacks turn out to be the oscillation and bistability generators while the global ones prolong the oscillation. In the JAK/STAT signaling, one local and one global feedback are intertwined to adjust the dynamic pattern. On the other hand, such control strategy may as well speed up evolution of signaling networks since new biological functions could emerge from the mutation changing connections between well conserved feedbacks and forward modules or starting new crosstalks between existing feedback controllers. Meanwhile, a mutation that substantially changes local feedback connections is quite unlikely for two reasons: first, the number of involved nodes is small and the probability of random mutation is small; second, any unbalance in the control would significantly decrease the survival rate of such cells. This is probably the reason why many local feedback modules are well conserved during evolution. On the other hand, structure variation affects dynamical behavior of signaling networks in a context-dependent manner. Structures designed as such are utilized efficiently by cells in different tissues to give different responses for diversified signals. For example, different time scales of the ERK-PP oscillation or activation may induce different activities in human breast adenocarcinoma MCF-7 cells or carcinoma SCC-12F cells [47, 48]. Likewise, different mammalian cells take advantage of disparate feedback loops in the calcium signaling to generate desired responses [30]. We are not discussing here how cells decode different temporal dynamics. For more informations on this matter, see a recent review [40] by Purvis *et al*.

We take advantage of the decomposed network to locate converging nodes in the forward pathway of the MEPU or junctions between the forward and feedback modules. In most cases they are reaction hubs of the signaling network and are highly regulated. Probing these reaction hubs in signaling networks bears much significance in pharmacology [1] and reverse engineering of biological systems [49]. Reaction hubs may also coordinate activity of feedback modules. In order to efficiently control signaling events, feedback modules are more likely to act on major nodes such as target proteins. Local feedbacks directly participate in the regulation of fast chemical reactions while the global ones, in most case, attach to reaction hubs. As shown in Fig 1A, the global feedbacks Module 4 and 5 regulate the reactions producing the target protein IP3 (node 44) and the reaction hub G*βγ* (node 17). In the MAPK (Fig 1B) network, two global feedbacks act on the most regulated reaction hubs: (EGF-EGFR*)_2_-GAP-Grb2-Sos and (EGF-EGFR*)_2_-GAP-Shc*-Grb2-Sos. In the JAK/STAT signaling, the global feedbacks activate STAT1c to get STAT1c*. As previously discussed, concentration of target proteins is very sensitive to the alteration of kinetic parameters related to the reaction hubs. It seems that feedback modules act on specific points of a network. Since the behavior of the system is greatly controlled by these points, this type of regulation may be the major cooperation mechanism between feedback modules. Our decomposition and simulation analysis also offers an intriguing glimpse of how degeneracy and robustness is achieved in complex cell signaling systems. Cells tend to employ different redundancy strategies throughout the whole network to ensure the robustness of dynamics. Feedback modules acting on same signal forwarding paths are likely to embed functional redundancy, while even those controlling disparate signaling processes may have similar control over output signals. In the forward module, a set of pathways linking the same endpoints often have similar influences on the target protein manifesting their redundancy in relaying signals. Hierarchical feedback modules and functionally redundant structures bestow cells capacity of stabilizing signal transduction processes and coordinate their activities with environmental changes [36].

## Conclusion

In this paper, based on the underlying information flow, we analyzed in great detail three different cell signaling systems: the GPCR, the MAPK and the JAK/STAT network employing a global top-down graph theoretic algorithm coupled with dynamics consideration, which shows its strength in dealing with complex signaling networks. The forward and feedback modules of each network are identified and their detailed functions are tested and explained. Our result exhibits the detailed mechanism of how feedback modules cooperatively maintain the versatility and robustness in signaling [50]. Moreover, hierarchical structure of feedbacks are observed not only in network topology but also in the dynamic regulation as revealed by module perturbation. Global feedbacks wrap the local ones in the three cell signaling networks and our analysis is able to systematically unfold different functions of each modulating unit, producing an onion-like temporal profiles. Moreover, multiple paths in the forward module and the controls exerted by different types of feedback modules give rise to functional redundancy and leads to the emergence of reaction hubs.

The current decomposition of networks reduces difficulties in analyzing complex signaling systems and provides new perspectives to probe the relationship between network structure and their diversified function. The verified design principles will provide insights for reverse engineering of biochemical systems [49], which find wide application in synthetic biology [51], drug design, disease prognosis and so forth. The findings here also help revealing the evolutionary features of signaling networks and uncover versatility and robustness associated to biological system. Nevertheless, many problems and questions remain to be solved or answered, for example, how organized feedback modules influence the spread of omnipresent noise, how distributed feedbacks are responsible for the parameter sensitivity or insensitivity, what are the basic topological patterns underlying multifarious feedback loops [52]. These challenges ask for better study of dynamics and functions of cell life activity, which requires close collaboration between experimentalists and theorists.

## Materials and Methods

### Procedures in the decomposition of signaling networks

#### 1. Build a network depicting all chemical reactions

**Fig 6:**
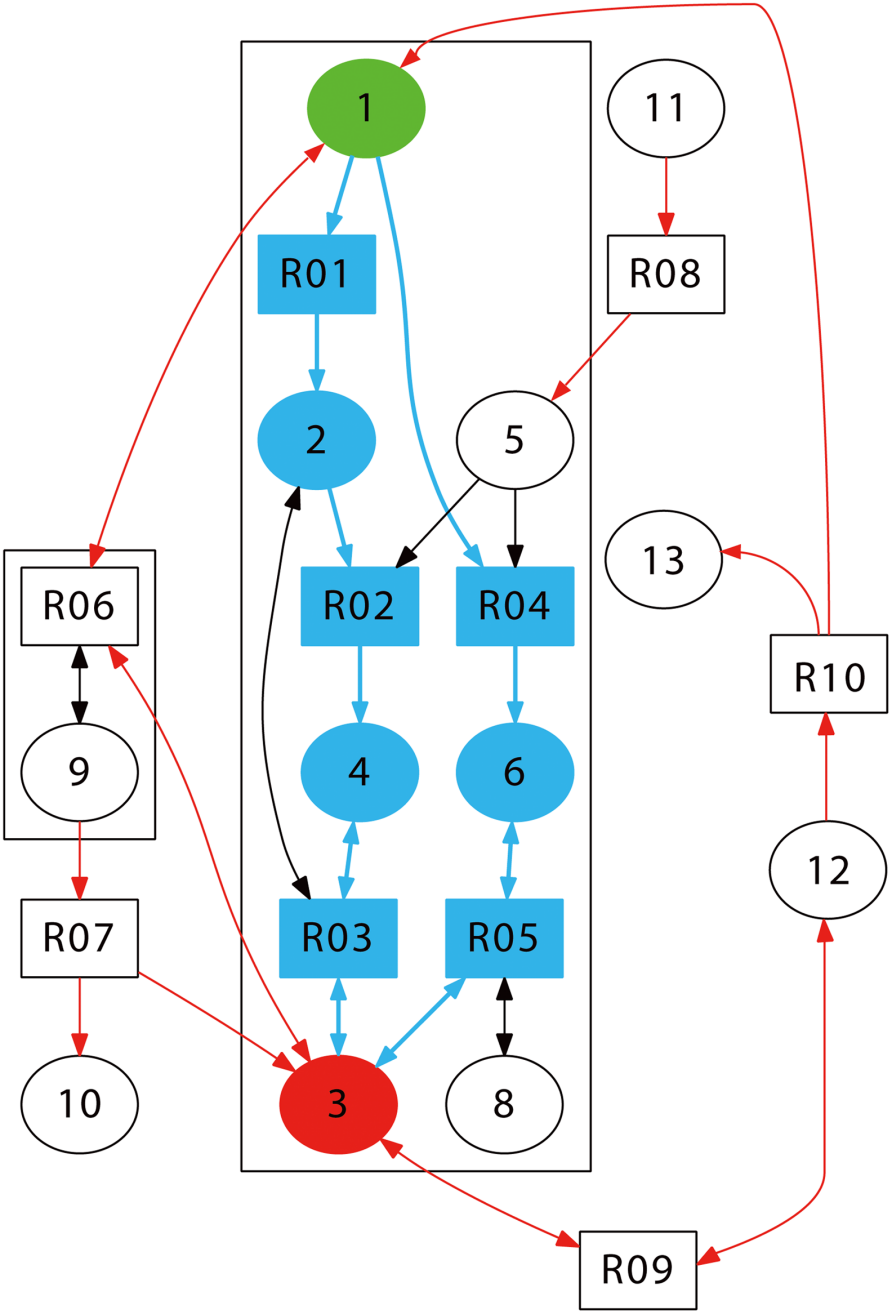
The toy model employed in the method for illustrating of our method.

First of all, a network of chemical reactions involved in a specific cell signaling process is constructed. In our scenario, reactions catalyzed by enzymes are assumed to be of Michaelis-Menten type and consequently represented by a synthesis reaction followed by a dissociative one. A toy model (Fig 6) including 9 reactions in Table 1 is taken as an example. Nodes in the graph (Fig 6) have two shapes. The square nodes represent reactions and the round ones stand for reactants or products. The edge 1– > R01 means that reactant 1 participates in reaction R01, and the edge R01– > 2 means that product 2 is generated through reaction R01. Meanwhile, the edge associated to one reaction is labeled with a positive integer while the edge for the reverse with the negative. Table 2 is a list of edges in this network. S and E represent the start and the end of each edge and numbers in italics are indices. Taken as a whole, the network is polarized with given input and output.

**Table 1:** Edges in the toy model.

Node	Reaction	Node	Reaction
R01	[1] —> [2]	R06	[3] + [1] <—> [9]
R02	[5] + [2] —> [4]	R07	[9] —> [10] + [3]
R03	[4] <—> [2] + [3]	R08	[11] —> [5]
R04	[1] + [5] —> [6]	R09	[3] <—> [12]
R05	[6] + [8] <—> [3]	R10	[12] —> [13] + [1]

**Table 2:** Edges in the toy model.

S	E	I	S	E	I	S	E	I	S	E	I	S	E	I	S	E	I
1	R01	*1*	2	R03	*-3*	6	R05	*5*	R06	3	*-6*	R07	3	*7*	12	R10	*10*
R01	2	*1*	R03	2	*3*	R05	6	*-5*	1	R06	*6*	11	R08	*8*	R10	13	*10*
5	R02	*2*	3	R03	*-3*	8	R05	*5*	R06	1	*-6*	R08	5	*8*	R10	1	*10*
2	R02	*2*	R03	3	*3*	R05	8	*-5*	9	R06	*-6*	3	R09	*9*			
R02	4	*2*	1	R04	*4*	3	R05	*-5*	R06	9	*6*	R09	3	*-9*			
4	R03	*3*	5	R04	*4*	R05	3	*5*	9	R07	*7*	12	R09	*-9*			
R03	4	*-3*	R04	6	*4*	3	R06	*6*	R07	10	*7*	R09	12	*9*			

S and E represent the start and the end of each edge and numbers in italics are indices.

#### 2. Search for short cycles that cover most edges of the network

Searching for a set of shortest cycles that cover most edges is the starting point to get forward and feedback modules since a cycle in a polarized network usually suggests the existence of a feedback. We simplify the problem by searching for the shortest paths connecting the tail to the head of each edge. However, such shortest path needs to satisfy two constraints: (1) the labels of two connected edges shouldn’t be opposite to each other to avoid the back-forth traversing of a reversible reaction. For example, in the toy model, node 1 cannot connect with node 3 through the path 1—> R06—> 3; (2) meanwhile, all the reactants participating in upstream reactions should not reappear in the latter part of the cycle and vice versa. Before searching for a cycle running through a specific edge, the connection from the tail to the head is deleted to eliminate the cycle containing only two edges since such a 2-cycle is representing a reversible reaction. We use the breadth first search (BFS) [53] method to find the shortest paths. However, edges are utilized instead of nodes in the BFS procedure. For example, if we want to find a cycle covers the edge R01—> 2, we need to find a route from node 2 to node R01. Starting from node 2 in the BFS method, we first get node R02 and node R03. Because of the first constraint, R03 cannot reach node 3. Meanwhile, since R03 has been used, a pathway like 2—> R02—> 4—> R03—> 3—> R09—> 12—> R10—> 1—> R01 is excluded. If we instead use edges, in this case, although the edge 2—> R03 has been used, the edge 4—> R03 being able to reach node R03 has not. What’s more, the reason why the cycle 1—> R06—> 9—> R07—> 3—> R09—> 12—> R10 is not in Table 3 is that: before reached from R07, node 3 has already participated in R06 which is forbidden by the second constraint. Actually, as shown in the toy model, R06 is in a feedback module serving as a buffer for node 3.

**Table 3:** Reaction cycles in the toy model.

cycle length	Reaction cycle
4	R02 —> 4 —> R03 —> 2
4	R06 —> 9 —> R07 —> 3
8	R04 —> 6 —> R05 —> 3 —> R09 —> 12 —> R10 —> 1
10	R01 —> 2 —> R02 —> 4 —> R03 —> 3 —> R09 —> 12 —> R10 —> 1

In the current scheme, although not all edges are required to be covered, a set of shortest cycles that cover as many available edges as possible perform better than a complete set of cycle generators as in a previous work [18] where edges are undifferentiated and cycles are detected on a local scale.

#### 3. Determine the forward module

In order to achieve such distinction, we utilize the principle of minimal feedbacks first proposed in a previous work [18]. Briefly, the principle states that in a complex polarized cell signaling system, the number of edges in the forward module achieves should be maximized to operational efficiency and energy saving for cell signaling. Below details of searching for the forward paths are discussed which mainly include three steps:

i) In order to figure out how the final product is produced through a chain of reactions, we construct a shortest path linking the input to the output using the BFS method in procedure 2. An additional constraint is imposed to cater to chemistry. Specifically, reactions along the shortest path should be simple ones where all the reactants are present initially or generated from upstream reactions.

If the constraint cannot be satisfied we try to merge pathways to make through. As an example from the toy model, the pathway 1—> R01—> 2—> R02—> 4—> R03—> 3 does not seem to be viable if node 5 is not available initially (zero initial concentration). However, if node 11 is nonzero initially, its mergence with the route 11—> R08—> 5—> R02 makes a reasonable path.

ii) Searching for all paths connecting the input to the output. First, for cycles containing both the input and the output node, we get the first-generation paths. In the toy model two such pathways: 1—> R01—> 2—> R02—> 4—> R03—> 3 and 1– > R04—> 6—> R05—> 3, are found. In addition, the simple path obtained in the previous step should be placed into the first generation. Then we search every cycle obtained in Part 2 for nodes overlapping with any path in the first generation. The fragment between two most separated overlapping nodes of a cycle will replace the original fragment in the path to get a new second generation path. Repeating the procedure until no new edge could be recruited. Edges in these paths are the main part of the forward module.

iii) Moreover, all reactants in the multi-component reactions belonging to a specific path are also included in the forward module in order to relay signals by sequential chemical reactions from the input to the output.

#### 4. Determine the MEPU (most efficient production unit)

The paths we get in Part 3 constitute the forward module. To partially order chemicals in this subgraph we need to apply the HVD (horizontal and vertical decomposition) method [54]. The HVD method is able to extract the uni-directional layered structure of a directed network: upstream layers may influence downstream ones but not vice versa. In graph theory, finding all paths connecting two nodes in a strongly connected component is an NP hard problem. We simplify this problem by utilizing only segments of shortest cycles obtained in Part 2 to reduce computation load. In Part 3, searching stops if no new edges are found. Nonetheless, still a large number of paths are available. It turns out that a sizable number of nodes are placed in the same layer when we apply directly the HVD to this subgraph. As a result, the entanglement of the paths significantly impedes an ordered organization of chemical reactions in the forward module. In order to avoid this trouble, it is necessary to pick up a set of important paths considered to belong to the most efficient production unit (MEPU) built from the reactants whose initial concentration is non-zero. We expound the selection procedure in two parts:

i) Merging bypass into the forward pathway and prioritizing the paths in Part 2. Initially, all walkers are standing on the input node and begin to move forward until a reactant is found neither in the initial set nor in products of previous steps. Then we search for possible paths that could generate this reactant and hence are called bypasses. If more than one bypasses are found, the one employing the least type of initial reactants is taken. The choice is by no means unique but convenient for selecting and ranking paths in the MEPU. In order to achieve this, the cumulated reactants and products are recorded for each path. Once obtained, this bypass is merged to the pathway where the walker has been frozen and the walker moves again until no walker is able to make any progress. The faster a walker arrives at the output, the higher priority a pathway. This priority level will be used in latter steps.

ii) Selecting the MEPU in the forward module. First, the paths where the walker can’t reach the end are ruled out. The remaining paths are sorted according to the number of involved initial reactants in an ascending order. During this procedure, those with low priority and the same set of initial reactants are deleted. If the priorities yet remain the same, we keep the one with minimum path length. Afterwards, the sorted pathways are integrated one by one. If no new initial reactant is recruited in an integration, the corresponding path will not be included. The procedure terminated if all the initial reactants are covered.

The survived paths after the selection process constitute the MEPU of the network. This unit highly depends on the input signal, mainly because our method is aimed at safeguarding the independence of these paths and the efficiency in the utilization of initial reactants, using as few reactions as possible.

#### 5. Apply the HVD method to the MEPU and identify feedback networks

The HVD method is applied to the MEPU to partially order the edges and nodes in it. Based on the horizontal and vertical position of each node, the MEPU depict a detailed map of forward signaling. With the MEPU scooped out of the forward module, the remaining nodes and connections make a subgraph.

The nodes absent in the forward module are assigned to feedback modules. In the same way, the HVD method is applied to each connected part outside the forward module to get different feedback modules carrying out different signaling regulations.

#### 6. Decompose possible complicated feedback modules

If any of the feedback module remains complex. We define new inputs that collect information from downstream and new outputs feeding signals back to upstream in the forward module and kick off another decomposition of the module.

### Details and related assumptions of the HVD method

The HVD method can only be applied to a directed network. Above all, a transition matrix *T* which differs somewhat from an adjacency matrix *T*′ is constructed. The adjacency matrix element $T_{ij}^{\prime }=1$ signifies an edge in the network starting from the *i*-th node and ending at the *j*-th node and the rest elements $T_{ij}^{\prime }=0$. The transition matrix can be obtained through the adjacency matrix. Specifically, the diagonal elements $T_{ii}^{\prime }$ are set to be one and then each element in the same column are divided by the sum of the non-zero values in this column. Secondly, the left eigenvectors for eigenvalue 1 of the transition matrix *T* are added up to a new vector. The non-zero elements in the new vector correspond to the graph nodes in the bottom layer. Thirdly, delete the nodes in the bottom layer and all the linking edges to get a new graph. Return to the second step to get the upper layer until the size of the graph equals zero.

### All the Chemical reactions and reactants in the three signaling systems

All the chemical reactions and reactants related to the nodes in the three signaling systems (Fig 1) are listed in the supporting information. S1 and S3 Tables are for the GPCR signaling network, S4 and S6 Tables for the MAPK signaling system and S7 and S9 Tables for the JAK/STAT signaling pathway. The initial reactants for the GPCR, the MAPK and the JAK/STAT signaling network are respectively given in S2, S5 and S8 Tables. The kinetic parameters of the three signaling pathways are respectively given in the supporting information: S3, S6 and S9 Tables.

### Equations and parameters in the simulation

Stoichiometric coefficients [55, 56] are important description of chemical reactions, based on which the reaction rates can be inferred by the law of mass action. For a system with *N* chemical species and *R* reactions, the *j*-th reaction can be written in the form

$$\begin{array}{c} \sum_{i=1}^{N}s_{ij}X_{i}\overset{k_{j}}{\to }\sum_{i=1}^{N}r_{ij}X_{i}, \end{array}$$ (1)

where:

*j* is the reaction index of the *j*-th reaction, *X*_*i*_ is the *i*-th chemical species, *k*_*j*_ is rate coefficient of the *j*-th reaction and *s*_*ij*_ and *r*_*ij*_ are the stoichiometric constant. So the reaction rate is:

$$\begin{array}{c} \upsilon_{j}=k_{j}\prod_{i=1}^{N}{\left[X_{i}\right]}^{s_{ij}}. \end{array}$$ (2)

By defining the stoichiometric matrix:

$$\begin{array}{c} S_{ij}=r_{ij}-s_{ij}, \end{array}$$ (3)

the specific chemical species’ concentration can be determined by the ODE in the generic form:

$$\begin{array}{c} \frac{d[X_{i}]}{dt}=\sum_{j=1}^{R}S_{ij}\upsilon_{j}. \end{array}$$ (4)

However, for systems containing reactions with intermediate steps, e.g, the enzymatic reactions, the Michaelis-Menten kinetics should be included into the description. The reaction rate *υ* is then given by

$$\begin{array}{c} \upsilon =\frac{V_{\max}[S]}{K_{m}+[S]}, \end{array}$$ (5)

where:

*V*_max_ is the maximum reaction rate at maximum substrate concentration: [*S*]. The Michaelis constant *K*_*m*_ is the substrate concentration when the reaction rate is half of the *V*_max_. In the supplementary tables, such reactions are marked. The kinetic parameters and the initial concentration of chemical species are adopted from the literature [19, 23, 26]. For more details about the reactions and parameters, please see the supporting information: S3 Table for the GPCR signaling network, S6 Table for the MAPK signaling system and S9 Table for the JAK/STAT network.

### Code implementation and Graph plotting

The decomposition of signaling networks and the dynamics simulation is implemented in MATLAB. The graphs are plotted with Graphviz. Since we mainly order nodes in the MEPU as previously mentioned. The task to position the rest of nodes in the forward module is done by the graph-visualizing software Graphviz which is effective in most cases but couldn't guarantee every node is placed in the ideal position as the way we think of. That is another reason why schematic plots of signaling systems are presented. As an example, G*βγ*-GDP-G*α*q (N21) and G*βγ*-GDP-G*α*i (N15) are not placed in the higher layer (Fig 1A). If we enforce the right position of Node 4 and Node 8, a very messy graph is obtained. In the schematic plot of this signaling system (Fig 2A), G*βγ*-GDP-G*α*q and G*βγ*-GDP-G*α*i are placed in the right position.

## Supporting Information

S1 Fig The network model of the GPCR signaling system before decomposition. The network model constructed in the GPCR signaling system before decomposition. These nodes are randomly positioned. Two green nodes are the inputs of the network, while the red one represents the output node.103 nodes and 214 edges are involved in this complex network. The ovals symbolize reactants and products and squares functional nodes representing specific reactions. The edges connecting ovals to squares indicate the participants of each reaction, in addition, a bi-directional line indicates that the reaction is reversible.
(TIF)

S2 Fig The network model of the MAPK signaling system before decomposition. The network model of the MAPK signaling network. The green node is the input and the red one is the output. There are 121 nodes and 313 edges in the whole network.
(TIF)

S1 Table The name of each reactant in the GPCR network. (DOCX)

S2 Table Non-zero initial concentrations reactants in the GPCR signaling. (DOCX)

S3 Table All the chemical reactions involved in the GPCR signaling network. (DOCX)

S4 Table The name of each reactant in the MAPK signaling system. (DOCX)

S5 Table Non-zero initial concentration reactant in the MAPK signaling networks. (DOCX)

S6 Table All the chemical reactions involved in the MAPK signaling system. (DOCX)

S7 Table The name of each reactant in the JAK/STAT pathway. (DOCX)

S8 Table Non-zero initial concentrations reactants in the JAK/STAT signaling networks. (DOCX)

S9 Table All the chemical reactions involved in the JAK/STAT pathway. (DOCX)
