# Supplementary figures and images for: Hierarchical Feedback Modules and Reaction Hubs in Cell Signaling Networks
Source: PLoS One. 2015 May 7;10(5):e0125886. doi: 10.1371/journal.pone.0125886 (PMC4424001; doi:10.1371/journal.pone.0125886)

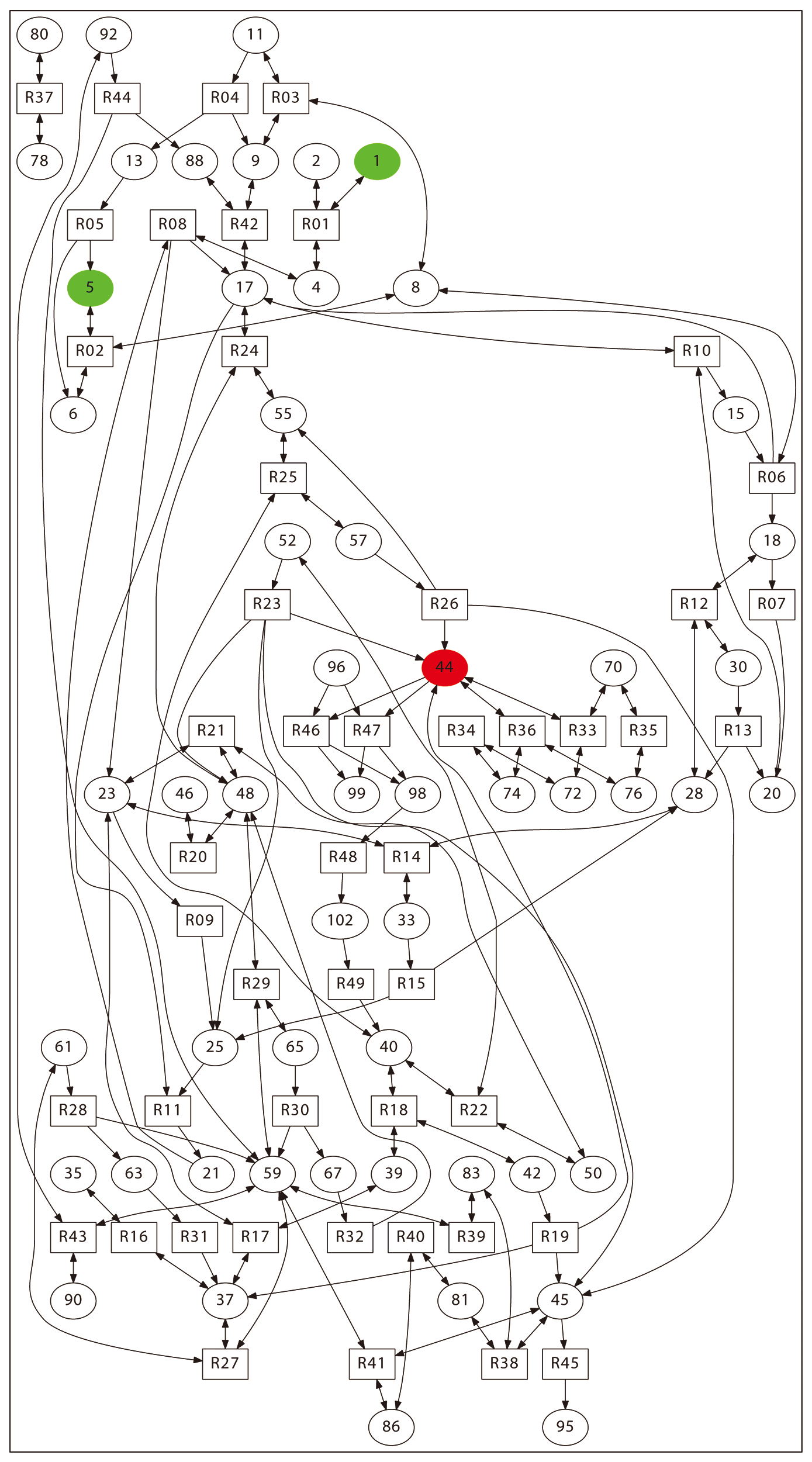

Supplement: S1 Fig — The network model constructed in the GPCR signaling system before decomposition. These nodes are randomly positioned. Two green nodes are the inputs of the network, while the red one represents the output node.103 nodes and 214 edges are involved in this complex network. The ovals symbolize reactants and products and squares functional nodes representing specific reactions. The edges connecting ovals to squares indicate the participants of each reaction, in addition, a bi-directional line indicates that the reaction is reversible. (TIF) [file pone.0125886.s001.tif]

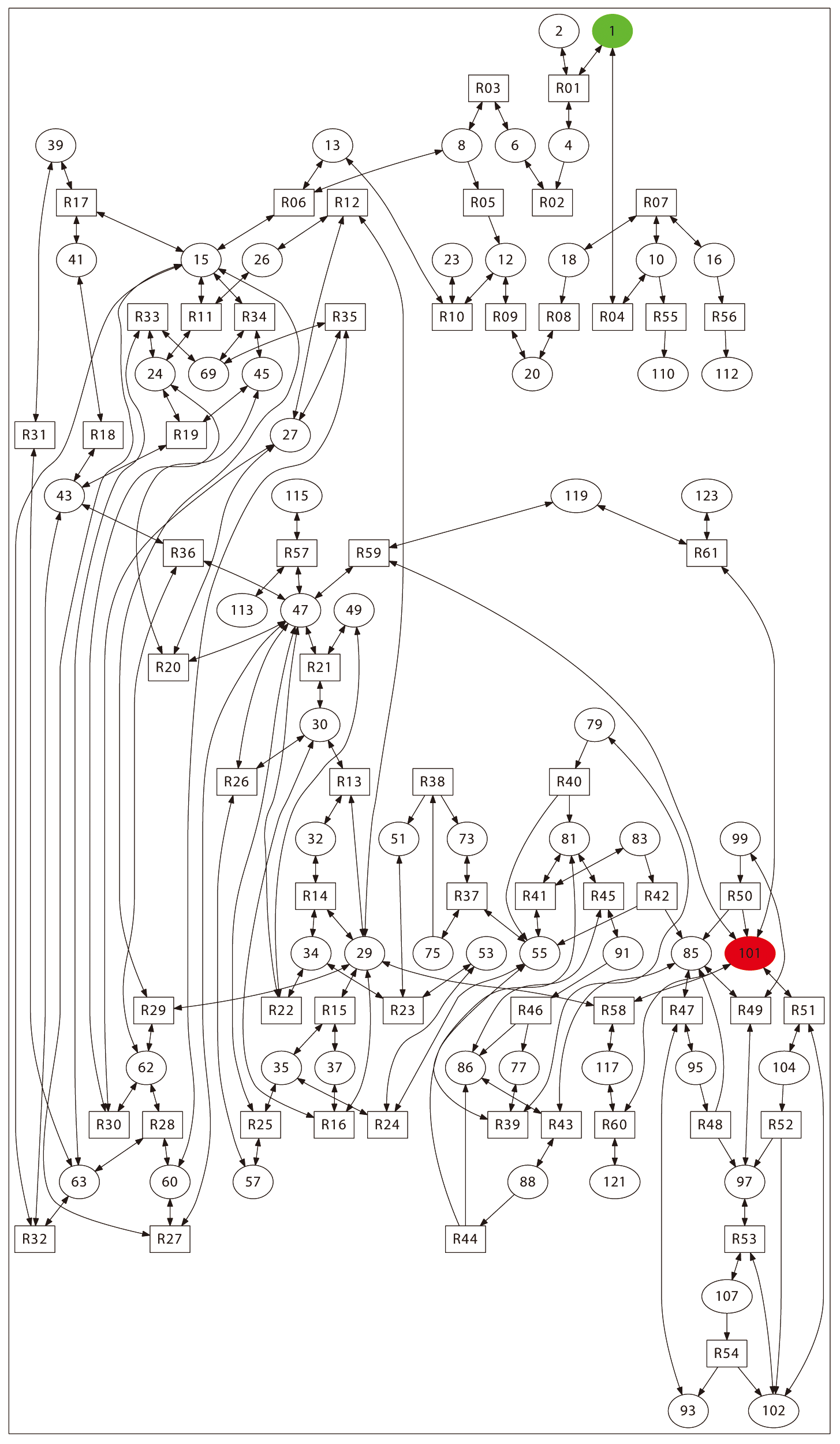

Supplement: S2 Fig — The network model of the MAPK signaling network. The green node is the input and the red one is the output. There are 121 nodes and 313 edges in the whole network. (TIF) [file pone.0125886.s002.tif]
