# Supplementary material for: Hierarchical Feedback Modules and Reaction Hubs in Cell Signaling Networks
Source: PLoS One. 2015 May 7;10(5):e0125886. doi: 10.1371/journal.pone.0125886 (PMC4424001; doi:10.1371/journal.pone.0125886)
Supplement: S1 Table — (DOCX) [file pone.0125886.s003.docx]

**S1 Table**

**The name of each reactant in the GPCR network.**

| Index^1^ | Reactant | Index | Reactant | Index | Reactant |
| --- | --- | --- | --- | --- | --- |
| N_1 | UDP | N_33 | RGS-a-Gαq-GTP | N_67 | PLCβ3-Ca-p |
| N_2 | p2yr | N_35 | PLCβ4 | N_70 | IP3R |
| N_4 | UDPC | N_37 | PLCβ4-Ca | N_72 | IP3R-IP3 |
| N_5 | c5a | N_39 | PLCβ4-Ca-Gαq-GTP | N_74 | IP3R-IP3-Ca |
| N_6 | c5aR | N_40 | PIP2 | N_76 | IP3R-Ca |
| N_8 | c5aC | N_42 | PLCβ4-Ca-Gαq-GTP-PIP2 | N_78 | Buf |
| N_9 | GRKp-Gβγ | N_44 | IP3 | N_80 | CaBuf |
| N_11 | GRKp-Gβγ-c5aC | N_45 | DAG | N_81 | PKC |
| N_13 | c5aCp | N_46 | PLCβ3 | N_83 | PKC-DAG |
| N_15 | Gβg-Gαi-GDP | N_48 | PLCβ3-Ca | N_86 | PKC-Ca |
| N_17 | Gβγ | N_50 | PLCβ3-Ca-Gαq-GTP | N_88 | GRKp |
| N_18 | Gαi-GTP | N_52 | PLCβ3-Ca-Gαq-GTP-PIP2 | N_90 | GRK |
| N_20 | Gαi-GDP | N_55 | PLCβ3-Ca-Gβγ | N_92 | PKC-DAG-Ca-GRK |
| N_21 | Gβγ-Gαq-GDP | N_57 | PLCβ3-Ca-Gβγ-PIP2 | N_95 | DAG-d |
| N_23 | Gαq-GTP | N_59 | PKC-DAG-Ca | N_96 | IP3K-a |
| N_25 | Gαq-GDP | N_61 | PKC-DAG-Ca-PLCβ4-Ca | N_98 | IP4 |
| N_28 | RGS-a | N_63 | PLCβ4-Ca-p | N_99 | IP3K-a |
| N_30 | RGS-a-Gαi-GTP | N_65 | PKC-DAG-Ca-PLCβ3-Ca | N_102 | IP5 |
| ^1^Members of the Index are corresponding to the nodes in Fig .1A. For example, ‘N_1’ is short for ‘Node 1’ and is related to the node with label ‘1’. | | | | | |
