# Supplementary material for: Hierarchical Feedback Modules and Reaction Hubs in Cell Signaling Networks
Source: PLoS One. 2015 May 7;10(5):e0125886. doi: 10.1371/journal.pone.0125886 (PMC4424001; doi:10.1371/journal.pone.0125886)
Supplement: S2 Table — (DOCX) [file pone.0125886.s004.docx]

**S2 Table**

**Non-zero initial concentrations reactants in the GPCR signaling networks**

| Components | Concentration * | Components | Concentration * | Components | Concentration * |
| --- | --- | --- | --- | --- | --- |
| c5aR | 5.00E-02 | IP3 | 1.80E-03 | PKC | 2.49E-02 |
| p2yr | 1.00E-01 | DAG | 1.00E-03 | GRK | 2.31E-02 |
| Gβγ | 7.14E+00 | IP3R | 2.08E-02 | RGS_a | 2.31E-02 |
| Gαi-GDP | 6.64E+00 | IP3R-IP3 | 1.75E-03 | Buf | 4.50E-01 |
| Gαq-GDP | 4.98E-01 | IP3R-IP3-Ca | 2.30E-03 | CaBuf | 5.05E-02 |
| PLCβ3 | 1.16E-01 | IP3R-Ca | 2.00E-04 | IP3K-a | 1.66E-03 |
| PLCβ4 | 6.64E-02 | Ca | 7.86E-02 | IP4 | 1.00E-01 |
| PIP2 | 5.00E-01 | CaER | 1.04E+01 | IP5 | 1.00E-01 |
| * (μM) | | | | | |
