# Supplementary material for: Hierarchical Feedback Modules and Reaction Hubs in Cell Signaling Networks
Source: PLoS One. 2015 May 7;10(5):e0125886. doi: 10.1371/journal.pone.0125886 (PMC4424001; doi:10.1371/journal.pone.0125886)
Supplement: S3 Table — (DOCX) [file pone.0125886.s005.docx]

**S3 Table**

**All the chemical reactions involved in the GPCR signaling network.**

| Node | Reaction | forward kinetic rate ** | reverse kinetic rate ** |
| --- | --- | --- | --- |
| R01 | [UDP] + [p2yr] <--> [UDPC] | 1.628 | 0.165 |
| R02 | [c5a] + [c5aR] <--> [c5aC] | 12.143 | 0.0378 |
| R03 | [GRKp-Gβγ] + [c5aC] <--> [GRKp-Gβγ-c5aC] | 591.54 | 12.367 |
| R04 | [GRKp-Gβγ-c5aC] --> [GRKp-Gβγ] + [c5aCp] | 123.31 | 0 |
| R05 | [c5aCp] --> [c5aR] + [c5a] | 0.0001 | 0 |
| R06 | [c5aC] + [Gβγ-Gαi-GDP] --> [c5aC] + [Gβγ] + [Gαi-GTP] | 0.0945 | 0 |
| R07 | [Gαi-GTP] --> [Gαi-GDP] | 0.0222 | 0 |
| R08 | [UDPC] + [Gβγ-Gαq-GDP] --> [UDPC] + [Gβγ] + [Gαq-GTP] | 0.2686 | 0 |
| R09 | [Gαq-GTP] --> [Gαq-GDP] | 0.0222 | 0 |
| R10 | [Gαi-GDP] + [Gβγ] --> [Gβγ-Gαi-GDP] | 7000 | 0 |
| R11 | [Gαq-GDP] + [Gβγ] --> [Gβγ-Gαq-GDP] | 7000 | 0 |
| R12 | [RGS-a] + [Gαi-GTP] <--> [RGS-a-Gαi-GTP] | 100 | 0.1 |
| R13 | [RGS-a-Gαi-GTP] --> [RGS-a] + [Gαi-GDP] | 100 | 0 |
| R14 | [RGS-a] + [Gαq-GTP] <--> [RGS-a-Gαq-GTP] | 100 | 0.1 |
| R15 | [RGS-a-Gαq-GTP] --> [RGS-a] + [Gαq-GDP] | 100 | 0 |
| R16 | [PLCβ4] + [Ca] <--> [PLCβ4-Ca] | 20 | 8 |
| R17 | [PLCβ4-Ca] + [Gαq-GTP] <--> [PLCβ4-Ca-Gαq-GTP] | 62.55 | 10.632 |
| R18 | [PLCβ4-Ca-Gαq-GTP] + [PIP2] <--> [PLCβ4-Ca-Gαq-GTP-PIP2] | 100 | 1 |
| R19 | [PLCβ4-Ca-Gαq-GTP-PIP2] --> [PLCβ4-Ca] + [Gαq-GDP] + [IP3] + [DAG] | 3 | 0 |
| R20 | [PLCβ3] + [Ca] <--> [PLCβ3-Ca] | 20 | 8 |
| R21 | [PLCβ3-Ca] + [Gαq-GTP] <--> [PLCβ3-Ca-Gαq-GTP] | 50 | 0.1 |
| R22 | [PLCβ3-Ca-Gαq-GTP] + [PIP2] <--> [PLCβ3-Ca-Gαq-GTP-PIP2] | 100 | 1 |
| R23 | [PLCβ3-Ca-Gαq-GTP-PIP2] --> [PLCβ3-Ca] + [Gαq-GDP] + [IP3] + [DAG] | 3 | 0 |
| R24 | [Gβγ] + [PLCβ3-Ca] <--> [PLCβ3-Ca-Gβγ] | 8.346 | 0.388 |
| R25 | [PLCβ3-Ca-Gβγ] + [PIP2] <--> [PLCβ3-Ca-Gβγ-PIP2] | 80 | 8 |
| R26 | [PLCβ3-Ca-Gβγ-PIP2] --> [PLCβ3-Ca-Gβγ] + [IP3] + [DAG] | 1 | 0 |
| R27 | [PKC-DAG-Ca] + [PLCβ4-Ca] <--> [PKC-DAG-Ca-PLCβ4-Ca] | 10 | 11 |
| R28 | [PKC-DAG-Ca-PLCβ4-Ca] --> [PKC-DAG-Ca] + [PLCβ4-Ca-p] | 1 | 0 |
| R29 | [PKC-DAG-Ca] + [PLCβ3-Ca] <--> [PKC-DAG-Ca-PLCβ3-Ca] | 110 | 11 |
| R30 | [PKC-DAG-Ca-PLCβ3-Ca] --> [PKC-DAG-Ca] + [PLCβ3-Ca-p] | 1 | 0 |
| R31 | [PLCβ4-Ca-p] --> [PLCβ4-Ca] | 0.12 | 0 |
| R32 | [PLCβ3-Ca-p] --> [PLCβ3-Ca] | 0.12 | 0 |
| R33 | [IP3R] + [IP3] <--> [IP3R-IP3] | 177.47 | 2.2 |
| R34 | [IP3R-IP3] + [Ca] <--> [IP3R-IP3-Ca] | 0.411 | 0.0434 |
| R35 | [IP3R] + [Ca] <--> [IP3R-Ca] | 0.9 | 0.806 |
| R36 | [IP3R-Ca] + [IP3] <--> [IP3R-IP3-Ca] | 20 | 0.029 |
| R37 | [Ca] + [Buf] <--> [CaBuf] | 10 | 7 |
| R38 | [PKC] + [DAG] <--> [PKC-DAG] | 100 | 0.05 |
| R39 | [PKC-DAG] + [Ca] <--> [PKC-DAG-Ca] | 10 | 6 |
| R40 | [PKC] + [Ca] <--> [PKC-Ca] | 0.01 | 30 |
| R41 | [PKC-Ca] + [DAG] <--> [PKC-DAG-Ca] | 1000 | 0.0001 |
| R42 | [GRKp] + [Gβγ] <--> [GRKp-Gβγ] | 1 | 0.05 |
| R43 | [PKC-DAG-Ca] + [GRK] <--> [PKC-DAG-Ca-GRK] | 158.49 | 10 |
| R44 | [PKC-DAG-Ca-GRK] --> [PKC-DAG-Ca] + [GRKp] | 10 | 0 |
| R45 | [DAG] --> [DAG-d] | 0.35 | 0 |
| R46 | [IP3] + [IP3K-a] --> [IP4] + [IP3K-a ] | V_IP3-IP4_^1^ | 0 |
| R47 | [IP3] + [IP3K-a] --> [IP4] + [IP3K-a] | K_IP3-IP4_^1^ | 0 |
| R48 | [IP4] --> [IP5] | V_IP4-IP5_/K_IP4-IP5_^2^ | 0 |
| R49 | [IP5] --> [PIP2] | 0.008 | 0 |
| The red [Ca] is removed during decomposing the signaling network, but still added into the system for dynamic simulation.  This model also contains additional processes, such as the Ca uptake and leak:  Ratio of ER volume/cell: de young: 0.185  Ca channel flux constant: 1E8 s-1  Leak flux constant: 0.15 s-1  Maximum Ca uptake rate (SERCA): 20 μM s-1  Activation constant of SERCA pump: 0.65 μM  Ca leak into the cell from outside: 0.0055 μM s-1  Na/Ca exchange activation const: 0.25 μM  Maximum Ca exchange rate: 0.023 μM s-1  ^1^ V_IP3-IP4_ = 13.9 s^-1^ , K_IP3-IP4_ = 0.055 μM (reaction obey the Michaelis-Menten equation)  ^2^ V_IP4-IP5_ = 100 μM s-1 K_IP4-IP_ = 1.4 μM (reaction obey the Michaelis-Menten equation)  ** first order rate constants in s-1 and second order rate constants in [uM-1 s-1]  All the simulation parameters are obtained from [1]. More information can be found in the model proposed by Flaherty P *et al* [1].  1. Flaherty P, Radhakrishnan ML, Dinh T, Rebres RA, Roach TI, et al. (2008) A dual receptor crosstalk model of G-protein-coupled signal transduction. PLoS computational biology 4: e1000185. | | | |
