# Supplementary material for: Hierarchical Feedback Modules and Reaction Hubs in Cell Signaling Networks
Source: PLoS One. 2015 May 7;10(5):e0125886. doi: 10.1371/journal.pone.0125886 (PMC4424001; doi:10.1371/journal.pone.0125886)
Supplement: S4 Table — (DOCX) [file pone.0125886.s006.docx]

**S4 Table**

**The name of each reactant in the MAPK signaling system.**

| Index | Name |
| --- | --- |
| N_1^1^ | EGFR |
| N_2 | EGF |
| N_4 | EGF-EGFR |
| N_6 | (EGF-EGFR)2 |
| N_8 | (EGF-EGFR*)2 |
| N_10 | EGFRi |
| N_12 | (EGF-EGFRi*)2 |
| N_13 | GAP |
| N_15 | (EGF-EGFR*)2-GAP |
| N_16 | EGFi |
| N_18 | EGF-EGFRi |
| N_20 | (EGF-EGFRi)2 |
| N_23 | (EGF-EGFRi*)2-GAP |
| N_24 | Grb2 |
| N_26 | (EGF-EGFR*)2-GAP-Grb2 |
| N_27 | Sos |
| N_29 | (EGF-EGFR*)2-GAP-Grb2-Sos |
| N_30 | Ras-GDP |
| N_32 | (EGF-EGFR*)2-GAP-Grb2-Sos-Ras-GDP |
| N_34 | Ras-GTP |
| N_35 | Ras-GTP* |
| N_37 | (EGF-EGFR*)2-GAP-Grb2-Sos-Ras-GTP |
| N_39 | Shc |
| N_41 | (EGF-EGFR*)2-GAP-Shc |
| N_43 | (EGF-EGFR*)2-GAP-Shc* |
| N_45 | (EGF-EGFR*)2-GAP-Shc*-Grb2 |
| N_47 | (EGF-EGFR*)2-GAP-Shc*-Grb2-Sos |
| N_49 | (EGF-EGFR*)2-GAP-Shc*-Grb2-Sos-Ras-GDP |
| N_51 | Raf |
| N_53 | Raf-Ras-GTP |
| N_55 | Raf* |
| N_57 | (EGF-EGFR*)2-GAP-Shc*-Grb2-Sos-Ras-GTP |
| N_60 | Shc*-Grb2-Sos |
| N_62 | Grb2-Sos |
| N_63 | Shc* |
| N_69 | Shc*-Grb2 |
| N_73 | Phosphatase1 |
| N_75 | Raf*-Phosphatase1 |
| N_77 | MEK |
| N_79 | MEK-Raf* |
| N_81 | MEK-P |
| N_83 | MEK-P-Raf* |
| N_85 | MEK-PP |
| N_86 | Phosphatase2 |
| N_88 | MEK-PP-Phosphatase2 |
| N_91 | MEK-P-Phosphatase2 |
| N_93 | ERK |
| N_95 | ERK-MEK-PP |
| N_97 | ERK-P |
| N_99 | ERK-P-MEK-PP |
| N_101 | ERK-PP |
| N_102 | Phosphatase3 |
| N_104 | ERK-PP-Phosphatase3 |
| N_107 | ERK-P-Phosphatase3 |
| N_110 | EGFRideg |
| N_112 | EGFideg |
| N_113 | Prot |
| N_115 | (EGF-EGFR*)2-GAP-Shc*-Grb2-Sos-Prot |
| N_117 | (EGF-EGFR*)2-GAP-Grb2-Sos-ERK-PP |
| N_119 | (EGF-EGFR*)2-GAP-Shc*-Grb2-Sos-ERK-PP |
| N_121 | (EGF-EGFR*)2-GAP-Grb2-Sos-deg |
| N_123 | (EGF-EGFR*)2-GAP-Shc*-Grb2-Sos-deg |
| ^1^Members of the Index are corresponding to the nodes in Fig, 1B. For example, ‘N_1’ is short for ‘Node 1’ and is related to the node with label ‘1’. | |
