# Supplementary material for: Hierarchical Feedback Modules and Reaction Hubs in Cell Signaling Networks
Source: PLoS One. 2015 May 7;10(5):e0125886. doi: 10.1371/journal.pone.0125886 (PMC4424001; doi:10.1371/journal.pone.0125886)
Supplement: S5 Table — (DOCX) [file pone.0125886.s007.docx]

**S5 Table**

**Non-zero initial concentration reactant in the MAPK signaling networks.**

| Components | Concentration * | Components | Concentration * | Components | Concentration * |
| --- | --- | --- | --- | --- | --- |
| EGF | 5.00E-08 | Sos | 2.63E+04 | Phosphatase1 | 4.00E+04 |
| EGFR | 5.00E+04 | Ras-GDP | 7.20E+04 | MEK | 2.10E+07 |
| Prot | 8.10E+04 | Grb2-Sos | 4.00E+04 | phosphatase2 | 4.00E+04 |
| GAP | 1.20E+04 | Shc | 1.01E+05 | ERK | 2.21E+07 |
| Grb2 | 1.10E+04 | Raf | 4.00E+04 | phosphatase3 | 1.00E+07 |
| * in molecules per cell | | | | | |
