# Supplementary material for: Hierarchical Feedback Modules and Reaction Hubs in Cell Signaling Networks
Source: PLoS One. 2015 May 7;10(5):e0125886. doi: 10.1371/journal.pone.0125886 (PMC4424001; doi:10.1371/journal.pone.0125886)
Supplement: S6 Table — (DOCX) [file pone.0125886.s008.docx]

**S6 Table**

**All the chemical reactions involved in the MAPK signaling system.**

| Node | Reaction | forward Reaction rate** | reverse kinetic rate** |
| --- | --- | --- | --- |
| R01 | [EGFR]+[EGF] <--> [EGF-EGFR] | 30000000 | 0.0038 |
| R02 | [EGF-EGFR]+[EGF-EGFR] <--> [(EGF-EGFR)2] | 1.66E-05 | 0.1 |
| R03 | [(EGF-EGFR)2] <--> [(EGF-EGFR*)2] | 1 | 0.01 |
| R04 | [EGFR] <--> [EGFRi] | 0.0005 | 0.005 |
| R05 | [(EGF-EGFR*)2] <--> [(EGF-EGFRi*)2] | 0.0005 | 0.005 |
| R06 | [(EGF-EGFR*)2]+[GAP] <--> [(EGF-EGFR*)2-GAP] | 6.64E-06 | 0.2 |
| R07 | [EGFRi]+[EGFi] <--> [EGF-EGFRi] | 0.0543 | 0.011 |
| R08 | [EGF-EGFRi]+[EGF-EGFRi] <--> [(EGF-EGFRi)2] | 1.66E-05 | 0.1 |
| R09 | [(EGF-EGFRi)2] <--> [(EGF-EGFRi*)2] | 1 | 0.01 |
| R10 | [(EGF-EGFRi*)2]+ [GAP] <--> [(EGF-EGFRi*)2-GAP] | 6.64E-06 | 0.2 |
| R11 | [(EGF-EGFR*)2-GAP]+[Grb2] <--> [(EGF-EGFR*)2-GAP-Grb2] | 1.66E-05 | 0.275 |
| R12 | [(EGF-EGFR*)2-GAP-Grb2]+[Sos] <--> [(EGF-EGFR*)2-GAP-Grb2-Sos] | 1.66E-05 | 0.06 |
| R13 | [(EGF-EGFR*)2-GAP-Grb2-Sos]+[Ras-GDP] <--> [(EGF-EGFR*)2-GAP-Grb2-Sos-Ras-GDP] | 2.50E-05 | 38000 |
| R14 | [(EGF-EGFR*)2-GAP-Grb2-Sos-Ras-GDP] <--> [(EGF-EGFR*)2-GAP-Grb2-Sos]+[Ras-GTP] | 0.5 | 1.66E-07 |
| R15 | [Ras-GTP*]+[(EGF-EGFR*)2-GAP-Grb2-Sos] <--> [(EGF-EGFR*)2-GAP-Grb2-Sos-Ras-GTP] | 3.50E-06 | 0.4 |
| R16 | [(EGF-EGFR*)2-GAP-Grb2-Sos-Ras-GTP] <--> [(EGF-EGFR*)2-GAP-Grb2-Sos]+[Ras-GDP] | 0.023 | 3.66E-07 |
| R17 | [(EGF-EGFR*)2-GAP]+[Shc] <--> [(EGF-EGFR*)2-GAP-Shc] | 3.50E-05 | 0.1 |
| R18 | [(EGF-EGFR*)2-GAP-Shc] <--> [(EGF-EGFR*)2-GAP-Shc*] | 6 | 0.06 |
| R19 | [(EGF-EGFR*)2-GAP-Shc*]+[Grb2] <--> [(EGF-EGFR*)2-GAP-Shc*-Grb2] | 1.66E-05 | 0.55 |
| R20 | [(EGF-EGFR*)2-GAP-Shc*-Grb2]+[Sos] <--> [(EGF-EGFR*)2-GAP-Shc*-Grb2-Sos] | 1.66E-05 | 0.0214 |
| R21 | [(EGF-EGFR*)2-GAP-Shc*-Grb2-Sos]+[Ras-GDP] <--> [(EGF-EGFR*)2-GAP-Shc*-Grb2-Sos-Ras-GDP] | 2.50E-05 | 38000 |
| R22 | [(EGF-EGFR*)2-GAP-Shc*-Grb2-Sos-Ras-GDP] <--> [(EGF-EGFR*)2-GAP-Shc*-Grb2-Sos] + [Ras-GTP] | 0.5 | 1.66E-07 |
| R23 | [Raf]+[Ras-GTP] <--> [Raf-Ras-GTP] | 1.66E-06 | 0 |
| R24 | [Raf-Ras-GTP] <--> [Raf*]+[Ras-GTP*] | 1 | 0.0117 |
| R25 | [Ras-GTP*]+[(EGF-EGFR*)2-GAP-Shc*-Grb2-Sos] <--> [(EGF-EGFR*)2-GAP-Shc*-Grb2-Sos-Ras-GTP] | 3.50E-06 | 0.4 |
| R26 | [(EGF-EGFR*)2-GAP-Shc*-Grb2-Sos-Ras-GTP] <--> [(EGF-EGFR*)2-GAP-Shc*-Grb2-Sos]+[Ras-GDP] | 0.023 | 3.66E-07 |
| R27 | [(EGF-EGFR*)2-GAP-Shc*-Grb2-Sos] <--> [(EGF-EGFR*)2-GAP]+[Shc*-Grb2-Sos] | 0.1 | 4.00E-07 |
| R28 | [Shc*-Grb2-Sos] <--> [Grb2-Sos]+[Shc*] | 0.2 | 3.50E-05 |
| R29 | [(EGF-EGFR*)2-GAP-Grb2-Sos] <--> [(EGF-EGFR*)2-GAP]+[Grb2-Sos] | 0.03 | 7.50E-06 |
| R30 | [Grb2-Sos] <--> [Grb2] +[Sos] | 0.0015 | 7.50E-06 |
| R31 | [Shc*] <--> [Shc] | 0.005 | 0 |
| R32 | [(EGF-EGFR*)2-GAP-Shc*] <--> [(EGF-EGFR*)2-GAP]+[Shc*] | 0.3 | 1.50E-06 |
| R33 | [Shc*]+[Grb2] <--> [Shc*-Grb2] | 1.66E-05 | 0.55 |
| R34 | [(EGF-EGFR*)2-GAP-Shc*-Grb2] <--> [(EGF-EGFR*)2-GAP]+[Shc*-Grb2] | 0.3 | 1.50E-06 |
| R35 | [Shc*-Grb2]+[Sos] <--> [Shc*-Grb2-Sos] | 5.00E-05 | 0.064 |
| R36 | [(EGF-EGFR*)2-GAP-Shc*] + [Grb2-Sos] <--> [(EGF-EGFR*)2-GAP-Shc*-Grb2-Sos] | 5.00E-05 | 0.0429 |
| R37 | [Raf*]+[Phosphatase1] <--> [Raf*-Phosphatase1] | 0.000118 | 0.2 |
| R38 | [Raf*-Phosphatase1] --> [Raf]+[Phosphatase1] | 1 | 0 |
| R39 | [MEK] + [Raf*] <--> [MEK-Raf*] | 0.195 | 0.033 |
| R40 | [MEK-Raf*] --> [MEK-P] +[Raf*] | 38.1 | 0 |
| R41 | [MEK-P]+[Raf*] <--> [MEK-P-Raf*] | 0.195 | 0.033 |
| R42 | [MEK-P-Raf*] --> [MEK-PP] + [Raf*] | 38.2 (38200) | 0 |
| R43 | [MEK-PP]+[Phosphatase2] <--> [MEK-PP-Phosphatase2] | 0.238 | 0.8 |
| R44 | [MEK-PP-Phosphatase2] --> [MEK-P] + [Phosphatase2] | 0.058 | 0 |
| R45 | [MEK-P]+[Phosphatase2] <--> [MEK-P-Phosphatase2] | 4.50E-07 | 0.5 |
| R46 | [MEK-P-Phosphatase2] --> [MEK]+[Phosphatase2] | 0.058 | 0 |
| R47 | [ERK]+[MEK-PP] <--> [ERK-MEK-PP] | 0.891 | 0.0183 |
| R48 | [ERK-MEK-PP] --> [ERK-P]+[MEK-PP] | 16 | 0 |
| R49 | [ERK-P]+[MEK-PP] <--> [ERK-P-MEK-PP] | 0.891 | 0.0183 |
| R50 | [ERK-P-MEK-PP] --> [ERK-PP]+[MEK-PP] | 10 | 0 |
| R51 | [ERK-PP]+[Phosphatase3] <--> [ERK-PP-Phosphatase3] | 2.35E-05 | 0.6 |
| R52 | [ERK-PP-Phosphatase3] --> [ERK-P]+[Phosphatase3] | 0.246 | 0 |
| R53 | [ERK-P] + [Phosphatase3] <--> [ERK-P-Phosphatase3] | 0.0833 | 0.5 |
| R54 | [ERK-P-Phosphatase3] --> [ERK]+[Phosphatase3] | 0.246 | 0 |
| R55 | [EGFRi] --> [EGFRideg] | 0.0055 | 0 |
| R56 | [EGFi] --> [EGFideg] | 0.00067 | 0 |
| R57 | [(EGF-EGFR*)2-GAP-Shc*-Grb2-Sos]+[Prot] <--> [(EGF-EGFR*)2-GAP-Shc*-Grb2-Sos-Prot] | 1.73E-07 | 0.00166 |
| R58 | [(EGF-EGFR*)2-GAP-Grb2-Sos]+[ERK-PP] <--> [(EGF-EGFR*)2-GAP-Grb2-Sos-ERK-PP] | 16.6 | 2 |
| R59 | [(EGF-EGFR*)2-GAP-Shc*-Grb2-Sos]+[ERK-PP] <--> [(EGF-EGFR*)2-GAP-Shc*-Grb2-Sos-ERK-PP] | 16.6 | 2 |
| R60 | [(EGF-EGFR*)2-GAP-Grb2-Sos-ERK-PP] <--> [(EGF-EGFR*)2-GAP-Grb2-Sos-deg]+[ERK-PP] | 10 | 0 |
| R61 | [(EGF-EGFR*)2-GAP-Shc*-Grb2-Sos-ERK-PP] <--> [(EGF-EGFR*)2-GAP-Shc*-Grb2-Sos-deg]+[ERK-PP] | 10 | 0 |
| ** first order rate constants in s-1 and second order rate constants in [M-1 s-1], almost all the parameters are taken form the model proposed by Hornberg *et al* [1]  1. Hornberg JJ, Binder B, Bruggeman FJ, Schoeberl B, Heinrich R, et al. (2005) Control of MAPK signalling: from complexity to what really matters. Oncogene 24: 5533-5542. | | | |
