# Supplementary material for: Hierarchical Feedback Modules and Reaction Hubs in Cell Signaling Networks
Source: PLoS One. 2015 May 7;10(5):e0125886. doi: 10.1371/journal.pone.0125886 (PMC4424001; doi:10.1371/journal.pone.0125886)
Supplement: S7 Table — (DOCX) [file pone.0125886.s009.docx]

**S7 Table**

**The name of each reactant in the JAK/STAT pathway.**

| N_1^1^ | R |
| --- | --- |
| N_2 | JAK |
| N_4 | R-JAK |
| N_5 | IFN |
| N_7 | IFN-R-JAK |
| N_9 | (IFN-R-JAK)2 |
| N_11 | (IFN-R-JAK*)2 |
| N_12 | STAT1c |
| N_14 | (IFN-R-JAK*)2-STAT1c |
| N_16 | STAT1c* |
| N_18 | (IFN-R-JAK*)2-STAT1c* |
| N_20 | (STAT1c*)2 |
| N_21 | SHP-2 |
| N_23 | (IFN-R-JAK*)2-SHP-2 |
| N_25 | PPX |
| N_27 | PPX-STAT1c* |
| N_30 | STAT1c-STAT1c* |
| N_32 | (STAT1n*)2 |
| N_33 | PPN |
| N_34 | STAT1n* |
| N_36 | PPN-STAT1n* |
| N_38 | STAT1n |
| N_41 | mRNAn |
| N_43 | mRNAc |
| N_45 | SOCS1 |
| N_47 | SOCS1-(IFN-R-JAK*)2 |
| N_49 | PPX-(STAT1c*)2 |
| N_53 | PPN-(STAT1n*)2 |
| N_55 | STAT1n-STAT1n* |
| N_58 | SOCS1-(IFN-R-JAK*)2-STAT1c |
| N_60 | SOCS1-(IFN-R-JAK*)2-STAT1c-SHP-2 |
| N_63 | (IFN-R-JAK*)2-STAT1c-SHP-2 |
| ^1^Members of the Index are corresponding to the nodes in Fig .1C. For example, ‘N_1’ is short for ‘Node 1’ and is related to the node with label ‘1’. | |
