# Supplementary material for: Hierarchical Feedback Modules and Reaction Hubs in Cell Signaling Networks
Source: PLoS One. 2015 May 7;10(5):e0125886. doi: 10.1371/journal.pone.0125886 (PMC4424001; doi:10.1371/journal.pone.0125886)
Supplement: S8 Table — (DOCX) [file pone.0125886.s010.docx]

**S8 Table**

**Non-zero initial concentrations reactants in the JAK/STAT signaling networks**

| Components | Concentration * | Components | Concentration * |
| --- | --- | --- | --- |
| STAT1c | 1000 | IFN | 1 |
| PPN | 60 | SHP-2 | 100 |
| JAK | 12 | PPX | 50 |
| R (Receptor) | 12 |  |  |
| * (nM) | | | |
