# Supplementary material for: Hierarchical Feedback Modules and Reaction Hubs in Cell Signaling Networks
Source: PLoS One. 2015 May 7;10(5):e0125886. doi: 10.1371/journal.pone.0125886 (PMC4424001; doi:10.1371/journal.pone.0125886)
Supplement: S9 Table — (DOCX) [file pone.0125886.s011.docx]

**S9 Table**

**All the chemical reactions involved in the JAK/STAT pathway.**

| Node | Reaction name | forward Reaction rate** | reverse kinetic rate** |
| --- | --- | --- | --- |
| R01 | [R]+[JAK]<-->[R-JAK] | 100 | 0.05 |
| R02 | [IFN]+[R-JAK]<-->[IFN-R-JAK] | 20 | 0.02 |
| R03 | [IFN-R-JAK]+[IFN-R-JAK]<-->[(IFN-R-JAK)2] | 40 | 0.2 |
| R04 | [(IFN-R-JAK)2]-->[(IFN-R-JAK*)2] | 0.005 | 0 |
| R05 | [(IFN-R-JAK*)2]+[STAT1c]<-->[(IFN-R-JAK*)2-STAT1c] | 8 | 0.8 |
| R06 | [(IFN-R-JAK*)2-STAT1c]-->[(IFN-R-JAK*)2]+[STAT1c*] | 0.4 | 0 |
| R07 | [(IFN-R-JAK*)2]+[STAT1c*]<-->[(IFN-R-JAK*)2-STAT1c*] | 5 | 0.5 |
| R08 | [STAT1c*]+[STAT1c*]<-->[(STAT1c*)2] | 20 | 0.1 |
| R09 | [(IFN-R-JAK*)2]+[SHP-2]<-->[(IFN-R-JAK*)2-SHP-2] | 1 | 0.2 |
| R10 | [(IFN-R-JAK*)2-SHP-2]-->[(IFN-R-JAK)2]+[SHP-2] | 0.003 | 0 |
| R11 | [PPX]+[STAT1c*]<-->[PPX-STAT1c*] | 1 | 0.2 |
| R12 | [PPX-STAT1c*]-->[PPX]+[STAT1c] | 0.003 | 0 |
| R13 | [STAT1c]+[STAT1c*]<-->[STAT1c-STAT1c*] | 0.0002 | 0.2 |
| R14 | [(STAT1c*)2]-->[(STAT1n*)2] | 0.005 | 0 |
| R15 | [PPN]+[STAT1n*]<-->[PPN-STAT1n*] | 1 | 0.2 |
| R16 | [PPN-STAT1n*]-->[PPN]+[STAT1n] | 0.005 | 0 |
| R17 | [STAT1n]-->[STAT1c] | 0.05 | 0 |
| R18 | [(STAT1n*)2] --> [mRNAn] | Vmax/K^­1^ | 0 |
| R19 | [mRNAn]-->[mRNAc] | 0.001 | 0 |
| R20 | [mRNAc]-->[SOCS1] | 0.01 | 0 |
| R21 | [SOCS1]+[(IFN-R-JAK*)2]<-->[SOCS1-(IFN-R-JAK*)2] | 20 | 0.1 |
| R22 | [PPX]+[(STAT1c*)2]<-->[PPX-(STAT1c*)2] | 1 | 0.2 |
| R23 | [PPX-(STAT1c*)2]-->[PPX]+[STAT1c-STAT1c*] | 0.003 | 0 |
| R24 | [STAT1n*]+[STAT1n*]<-->[(STAT1n*)2] | 5 | 0.5 |
| R25 | [PPN]+[(STAT1n*)2]<-->[PPN-(STAT1n*)2] | 1 | 0.2 |
| R26 | [PPN-(STAT1n*)2]-->[PPN]+[STAT1n-STAT1n*] | 0.005 | 0 |
| R27 | [STAT1n]+[STAT1n*]<-->[STAT1n-STAT1n*] | 0.0002 | 0.2 |
| R28 | [STAT1c]+[SOCS1-(IFN-R-JAK*)2]<-->[SOCS1-(IFN-R-JAK*)2-STAT1c] | 8 | 0.8 |
| R29 | [SHP-2]+[SOCS1-(IFN-R-JAK*)2-STAT1c]<-->[SOCS1-(IFN-R-JAK*)2-STAT1c-SHP-2] | 1 | 0.2 |
| R30 | [SOCS1-(IFN-R-JAK*)2-STAT1c-SHP-2]-->[SOCS1]+[(IFN-R-JAK)2]+[STAT1c]+[SHP-2] | 0.003 | 0 |
| R31 | [SOCS1-(IFN-R-JAK*)2-STAT1c-SHP-2] --> [(IFN-R-JAK*)2-STAT1c-SHP-2] +[SOCS1] | 0.0005 | 0 |
| SOCS1 and mRNAn degrading rate: 0.0005 nM/s. These procedures are removed during decomposing the signaling network, but still added into the system for dynamic simulation.  ^1^ The transcription and translation process are modeled by the Michaelis-Menten equation where the Vmax = 0.01  nM/s and the K=400nM.  ** first order rate constants in s-1 and second order rate constants in [nM-1 s-1]  All the parameters are taken form the model proposed by Yamada S *et al* [1].  1. Yamada S, Shiono S, Joo A, Yoshimura A (2003) Control mechanism of JAK/STAT signal transduction pathway. FEBS letters 534: 190-196. | | | |
